# Systematic Study on Nonlinear Optical Chromophores with Improved Electro-Optic Activity by Introducing 3,5-Bis(trifluoromethyl)benzene Derivative Isolation Groups into the Bridge

**DOI:** 10.3390/molecules28020488

**Published:** 2023-01-04

**Authors:** Tongtong Liu, Fuyang Huo, Changqing Ge, Ya Li, Jing He, Han Zheng, Qian He, Yinsen Zhao, Zhuo Chen, Shuhui Bo

**Affiliations:** 1 Optoelectronics Research Centre, School of Science, Minzu University of China, Beijing 100081, China; 2 Engineering Research Centre of Photonic Design Software, Ministry of Education, Beijing 100081, China; 3 Key Laboratory of Bio-Inspired Materials and Interfacial Science, Technical Institute of Physics and Chemistry, Chinese Academy of Sciences, Beijing 100190, China

**Keywords:** nonlinear optical materials, organic electro-optic materials, electro-optic coefficient, isolation group, chromophore

## Abstract

A series of novel chromophores A, B, C, and D, based on the julolidinyl donor and the tricyanofuran (TCF) and CF_3_-tricyanofuran (CF_3_-Ph-TCF) acceptors, have been synthesized and systematically investigated. The *3,5*-bis(trifluoromethyl)benzene derivative isolation group was introduced into the bridge in the chromophores C and D. These nonlinear optical chromophores showed good thermal stability, and their decomposition temperatures were all above 220 °C. Density functional theory (DFT) was used to calculate the energy gaps and first-order hyperpolarizability (β). The macroscopic electro-optic (EO) activity was measured using a simple reflection method. The highest EO coefficient of poled films containing 35 wt% of chromophore D doped in amorphous polycarbonate afforded values of 54 pm/V at 1310 nm. The results indicate that the *3,5*-bis(trifluoromethyl)benzene isolation group can suppress the dipole–dipole interaction of chromophores. The moderate $r_{33}$ value, good thermal stability, and good yield of chromophores suggest their potential use in the nonlinear optical area.

## 1. Introduction

With the rapid development of science and technology, the photon has gradually replaced the electron as the carrier of information transmission, since it has a high transmission speed. High-performance electro-optic (EO) modulators are key components and can realize the photoelectric transformation for the boundaries of optical communication, computing, sensor technology, and ultra-broadband signal processing at GHz-THz bandwidths [1,2,3,4,5,6].

There are many kinds of materials for electro-optic modulators, such as III-V semiconductor (electrical absorption materials, e.g., GaAs, InP) [7], lithium niobite [8,9,10], 2D materials [11], and organic electro-optic (OEO) materials [12,13,14,15,16,17]. Among them, only lithium niobate and organic EO polymer are based on the Pockels effect. Compared to the lithium niobate, organic electro-optic polymers show large nonlinear optical coefficients, design diversity, fast response speed, and low cost [18,19]. Organic electro-optic polymers have great potential in the fields of Terahertz [20], electro-optic modulators, and other technical fields. In order to meet the application requirements of these technologies, organic electro-optic materials need to have a large EO coefficient ($r_{33}$), robust thermal stability, and good chemical stability. Through rational chromophore design and supra-molecular engineering such as molecular self-assembly and binary chromophore containing dendrimer glasses and polymers, the properties of organic electro-optic polymer materials have been greatly improved [21,22,23,24,25].

Typical organic electro-optic materials usually have a π electron conjugated system, which is usually composed of an electron donor, an electron acceptor, and a π-conjugated bridge. The nonlinear optical properties of the chromophore are related to the molecular first hyperpolarize ability (β). The first step is to design and synthesize chromophore molecules with large β values and high poling efficiency in the polymer to obtain large electro-optic activity. The molecular structure relationship shows that the stronger the electron push–pull ability of the electron donor and acceptor, the more obvious the charge transfer and the larger the -β value. In some organic electro-optic polymers, chromophore moieties with rod-like structures and strong dipole–dipole interactions result in antiparallel packing of chromophores. Therefore, the strength of donor, bridge, and acceptor groups can be modified, thus increasing the -β value. Different kinds of donors, acceptors, and bridges have been extensively studied to enrich the understanding of essential structure-nonlinear optical property and thermal stability correlations [26,27,28,29]. The introduction of some isolation groups (IG) into the chromophore moieties should be an efficient approach to decrease the interactions between chromophores and to enhance the nonlinear optical properties and the poling efficiency of organic electro-optic polymer materials [30,31,32]. These chromophores improve the application of nonlinear small molecules in the field of optical limiting and all-optical switching [33]. Future perspectives of the application of these kinds of NLO chromophores should be considered.

**Scheme 1:**
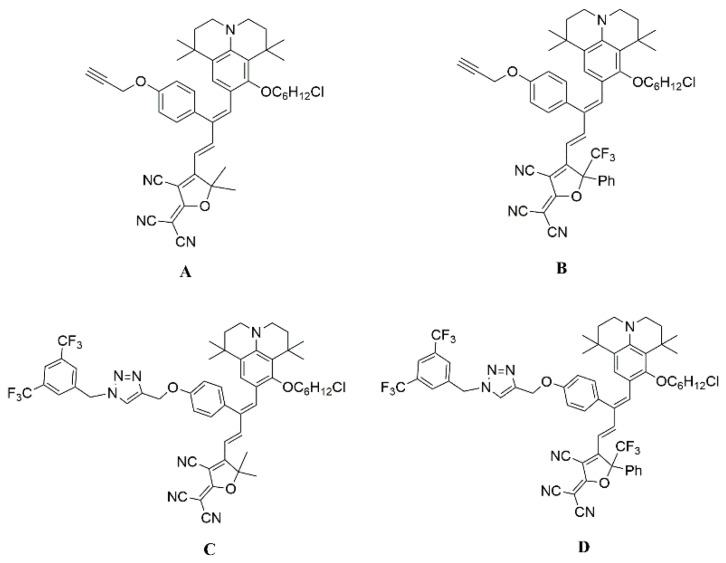
The chemical structures of chromophores A, B, C, and D.

In this work, the julolidinyl donor is used as the electron donor, and the tricyanofuran (TCF) and CF_3_-tricyanofuran (CF_3_-Ph-TCF) acceptors are used as the electron acceptors, which have super electron-withdrawing abilities. Furthermore, the *3,5*-bis(trifluoromethyl)benzene-based isolation group can also be introduced to modify a π-conjugated bridge using a Cu(I)-catalyzed click reaction to enhance the electron-donating ability and reduce the interaction between chromophores. Therefore, four chromophores with the julolidinyl donor and TCF or CF_3_-Ph-TCF acceptors were designed and synthesized, as shown in Scheme 1; the optimized structure of chromophore is closer to a spherical structure, which makes the rotation of dipole molecules more favorable under polarization voltage. The UV–Vis spectrum, solvatochromic behavior, thermal stability, DFT calculation, and EO activity of the four chromophores were systematically studied, and the structure–property relationships have been revealed.

## 2. Results and Discussion

### 2.1. Synthesis and Characterization

**Scheme 2:**
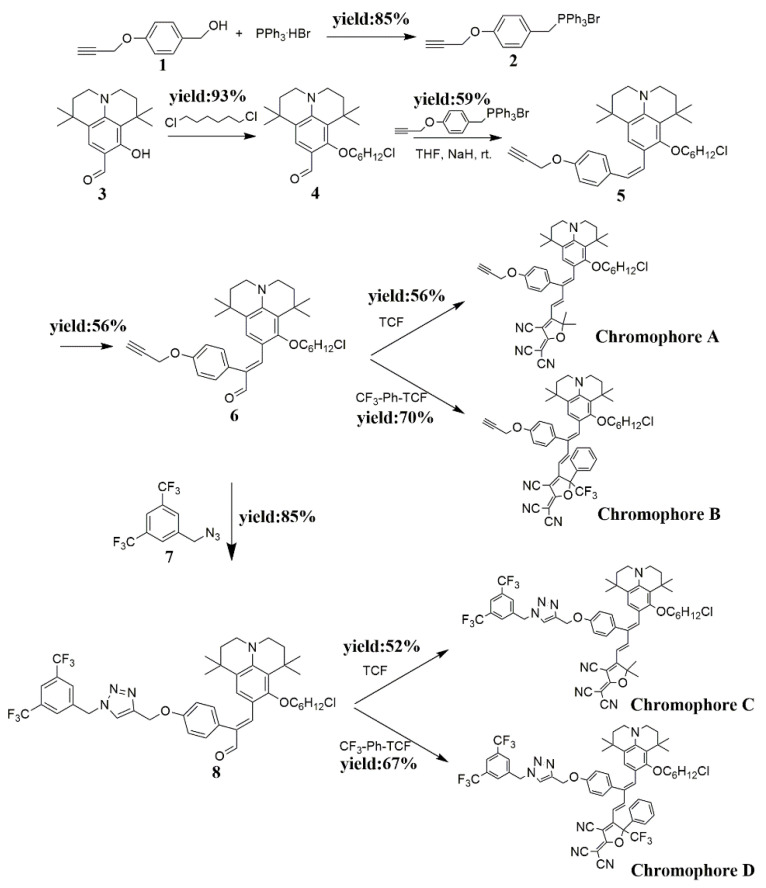
The synthesis route of chromophores A, B, C, and D.

As we all know, the 2-dicyanomethylene-3-cyano-4-methyl-2,5-dihydrofuran (TCF) acceptors with CF_3_ substituents CF_3_-Ph-TCF (2-dicyanomethylene-3-cyano-4-methyl-5-trifluorometheyl-5-(5-phenylthiophene-2-yl)-2,5-dihydrofuran) showed stronger electron-withdrawing ability, so the CF_3_-Ph-TCF was used to obtain the new materials with the higher electro-optic coefficient. The synthesis route of chromophores A, B, C, and D is presented in Scheme 2. These NLO chromophores were composed of the same julolidinyl electron donor, but they had different electron acceptors. Chromophores A and C had a strong electron acceptor, while chromophores B and D had an ultra-strong CF_3_-Ph-TCF electron acceptor. Compared with the chromophores A and B, chromophores C and D were designed to introduce the new long-branched chain (*3,5*-bis(trifluoromethyl)benzene) to the bridge of the chromophore using a Cu(I)-catalyzed click reaction. The flexible hindrance groups on π-conjugated bridges not only can increase the electron-withdrawing ability but also can reduce the interaction between chromophores.

### 2.2. Thermal Stability

**Figure 1:**
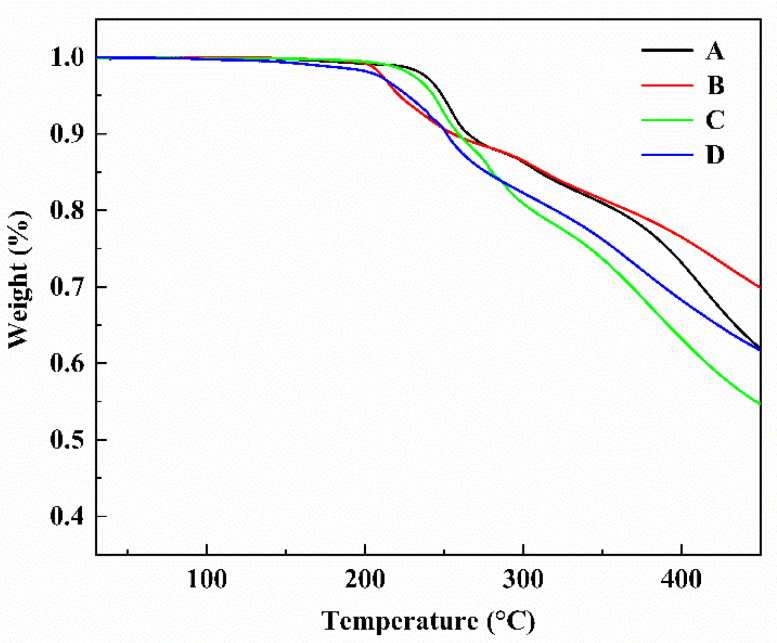
TGA curves of chromophores A–D with a heating rate of 10 °C·min^−1^ under nitrogen.

The electro-optic polymer material was poled under heating conditions to obtain an EO coefficient with good nonlinear properties. Therefore, NLO chromophores must be thermally stable enough to satisfy the temperature environment requirements (>200 °C) in electric field poling and subsequent processing of chromophores/polymer films. Thermal properties of the four chromophores were measured by thermogravimetric analysis (TGA) with a heating rate of 10 °C·min^−1^ under a nitrogen atmosphere. The temperature of weight loss was 5% corresponding to the decomposition temperature (T_d_) with the curve results as shown in Figure 1 and tabulated in Table 1. The T_d_ of chromophores A, B, C, and D are 249 °C, 221 °C, 243 °C, and 226 °C, respectively. The above results showed that the T_d_ of chromophores B and D with CF_3_-Ph-TCF was significantly lower than that of chromophores A and C with TCF acceptors because of the worse thermal stability of CF_3_-Ph-TCF acceptors than TCF. Among these chromophores, chromophore A shows the highest T_d_ of 249 °C indicating that the chromophores with the TCF acceptors but without the long chain are thermally robust. The above temperature (>220 °C) can meet the requirements of electric field poling near the glass transition temperature when the chromophores doped polymer materials.

**Figure 2:**
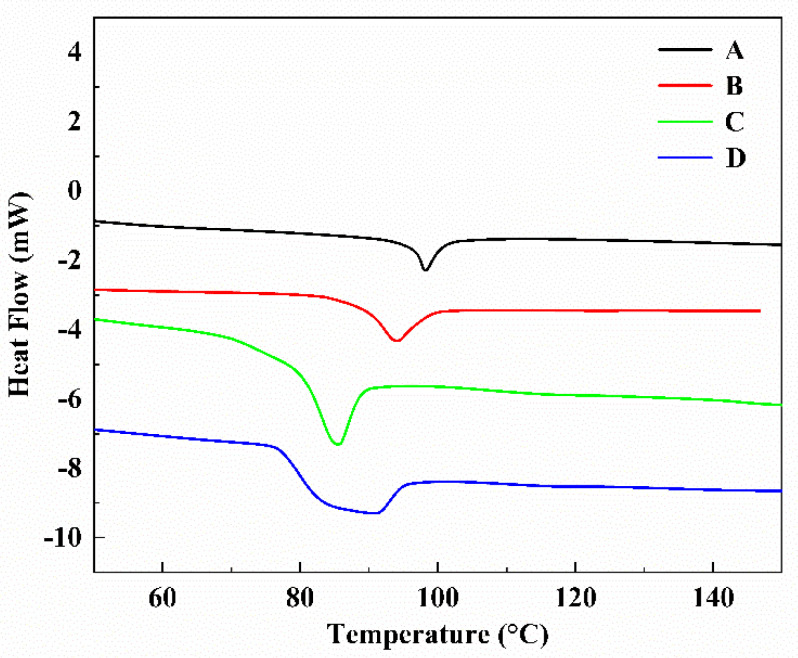
DSC curves of chromophores A–D under nitrogen at a heating rate of 10 °C·min^−1^.

In Figure 2, the glass transition temperature (T_g_) of the chromophores was measured by differential scanning calorimetry (DSC). The T_g_ of chromophores A and B are 98.2 °C and 90.4 °C, and the T_g_ of chromophores C and D are 83.5 °C and 80.2 °C, indicating the chromophores may be amorphous. The T_g_ of chromophores B and D are lower than the T_g_ of chromophores A and C because of CF_3_-Ph-TCF acceptors in the chromophores B and D. The T_g_ of chromophores C and D are lower than the glass transition temperatures T_g_ of chromophores A and B because of flexible hindrance groups in the chromophores C and D. The excellent thermal stability of the four chromophores makes them suitable for the fabrication of practical devices and electro-optical devices.

### 2.3. Optical Properties

**Figure 3:**
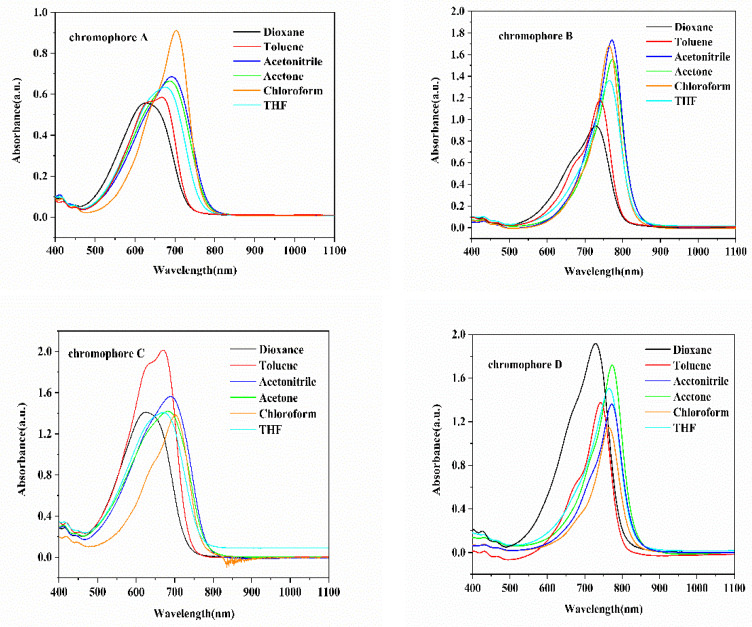
UV–Vis absorption spectra of chromophores A, B, C, D in six kinds of solvents with different dielectric constants (ε).

In order to reveal the influence of electron acceptors and electron bridge structures on intramolecular charge transfer (ICT), the UV–Vis absorption spectra of four chromophores were measured in a series of solvents with different dielectric constants, as shown in Figure 3 and Table 2. The solvatochromic behavior of the four chromophores can be investigated to explore the polarizability of the chromophores.

According to different structures, the absorption maxima (λ_max_) of chromophores A, B, C, and D are 702, 774, 702, and 774 nm in chloroform or acetone. Firstly, compared with chromophores A and C, chromophores B and D with the CF_3_-Ph-TCF acceptor showed larger red-shift maximum absorptions due to their stronger electron-withdrawing ability, which indicated that the introduction of a stronger acceptor can significantly improve ICT properties. Secondly, the absorption wavelength of chromophores C and D with the flexible hindrance group on the electron bridge was almost the same as chromophores A and B, indicating that the flexible hindrance group contributed almost nothing to the intramolecular charge transfer. In conclusion, the introduction of stronger acceptors can lead to better polarization for chromophores.

**Figure 4:**
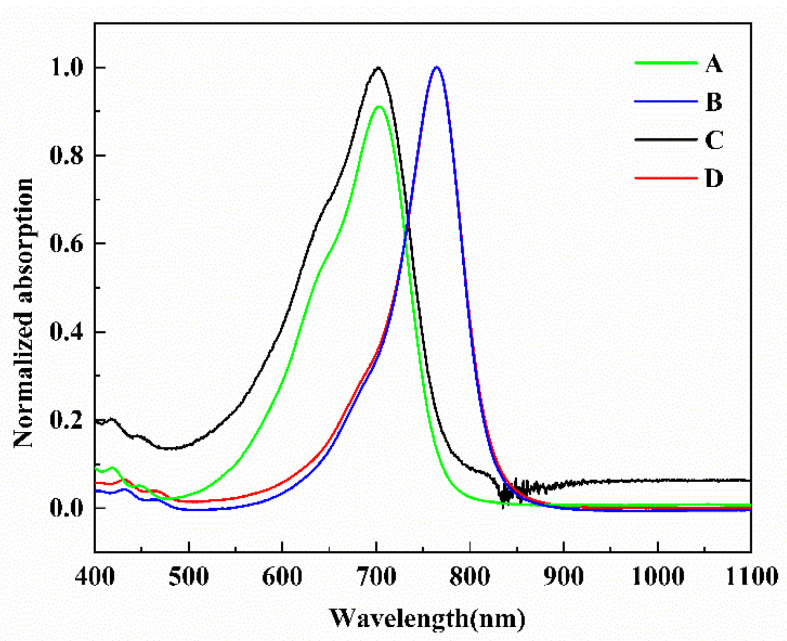
Normalized UV–Vis absorption spectrum of chromophores A, B, C, D in chloroform.

Because of the same acceptor TCF, the absorption spectra of chromophores A and C were similar, and the maximum absorption wavelength was measured in chloroform. The chromophores B and D had similar absorption spectra due to CF_3_-Ph-TCF acceptors, and the maximum absorption wavelength was measured in acetone. The minimum absorption wavelengths of the four chromophores were measured in dioxane. With the increase of the polarity of the solvent, the UV–Vis absorption initially exhibited a red shift and then appeared as a blue-shift and showed good solvation effect. Table 2, Δλ_max_ (the difference between λ_max_ in chloroform or acetone and λ_max_ in dioxane) shows a trend of B (44 nm) < D (46 nm) < A (77 nm) = C (77 nm), and the peak wavelength of chromophores A and C showed a bathochromic shift of 77 nm from dioxane to chloroform, suggesting that the chromophores A and C were more easily polarizable than the chromophores B and D, and they showed larger solvatochromism. The maximum absorption wavelength in chloroform was normalized, and the absorption spectra is shown in Figure 4.

### 2.4. Theoretical Calculations

In order to model the ground-state molecular geometries and understand the microscopic NLO properties of the designed chromophores, the HOMO–LUMO energy gaps and first-order hyperpolarizability β values of the four chromophores were calculated. The DFT calculations were carried out at the hybrid B3LYP level by employing the split valence 6-31+G* basis set using Gaussian 09. The data obtained from the DFT calculations are summarized in Table 3.

The frontier molecular orbitals are often used to characterize the chemical reactivity and kinetic stability of molecules, and they can obtain information about optical and electrical properties of molecules. The HOMO–LUMO energy gap is often used to understand the charge-transfer interaction that occurs in a chromophore molecule. The HOMO and LUMO energy of chromophores A, B, C, and D were calculated by DFT, and the energy gaps between the HOMO and LUMO energy for chromophores A and C were 4.01 eV and 4.06 eV, respectively. When the stronger CF_3_-Ph-TCF acceptor was introduced to the conjugated molecule, chromophores B and D were obtained, and their energy gaps were 3.95 eV and 3.99 eV, which were smaller than A and C. After introducing the flexible hindrance group to the electron bridge and CF_3_-Ph-TCF acceptor, the energy gaps (ΔE) showed a downtrend. As reported, the optical gap was lower, the ICT ability was higher, and the nonlinear performance was greater. The results showed that the optical bandgap of chromophores B and D is lower than that of chromophores A and C, which indicates that chromophores B and D should exhibit better ICT and NLO properties than chromophores A and C. As ΔE was reduced, chromophores B and D showed a bathochromic shift of λ_max_, and these results were consistent with the conclusions of the UV–Vis spectra analysis.

The frontier molecule orbitals of these chromophores are shown in Figure S1. The electron density is concentrated on the donor moiety at the HOMO state, but it is concentrated on the π-bridge and the acceptor moiety at the LUMO state. The comparison of the HOMO and LUMO electron distribution in the julolidinyl donor indicated easy delocalization of electrons in benzene rings. Consequently, both the hindrance groups can be treated as additional donors, which efficiently enhances the electron density of the conjugated system and increases the polarizability of chromophores.

In addition, the theoretical microscopic Zero frequency (static) molecular first hyperpolarizability (β) was calculated using Gaussian 09. As a reference reported earlier, β has been calculated at the CAM-B3LYP/6-31+G* level under vacuum. From this, the scalar quantity of β can be computed from the *x*, *y*, and *z* components according to the following equation:

$$\beta ={\left(\beta_{x}^{2}+\beta_{y}^{2}+\beta_{z}^{2}\right)}^{1∕2}$$

where

$$\beta_{i}=\beta_{iii}+\frac{1}{3}\underset{i\neq j}{\sum }\left(\beta_{\dot{i}jj}+\beta_{jij}+\beta_{jji}\right),i,j\in \left(x,y,z\right)$$

The data obtained from DFT calculations are summarized in Table 3. When used carefully and consistently, this method of DFT has been shown to give relatively consistent descriptions of first-order hyperpolarizability for a number of similar chromophores. The β values of chromophores A, B, C, and D were 225, 241, 243, and 255 × 10^−30^ esu, respectively. As reported earlier, the β value has a close relationship with the substituents, steric hindrance, intra-molecular charge transfer, π-conjugation length, and so on. The molecular quadratic hyperpolarizability (-β) values of CF_3_-Ph-TCF-based chromophores B and D were 7% and 5% larger than those of TCF-based chromophores A and C. Most remarkably, the extremely high β value (255 × 10^−30^ esu) was obtained by chromophore D, which possessed two favorable factors at the same time: the flexible hindrance group and the ultra-strong acceptor CF_3_-Ph-TCF. The λ_max_ of chromophores B and D were larger than that of chromophores A and C, so the trend of increasing β value was in good agreement with the trend of increasing λ_max_.

We optimized the structures of the chromophores to keep them in the most stable state using Gaussian 09 at the B3LYP/6-31g* level as shown in Figure S2. From the side views, we found that chromophores A and B without a flexible hindrance group on the electron bridge show good planarity, which may lead to serious dipole–dipole interactions, a barrier in noncentrosymmetric arrays. In addition, as for chromophores C and D, the flexible hindrance group occupied some steric hindrance; therefore, it kept chromophore molecules away from adjacent ones when poling, thus increasing the number of truly oriented chromophore molecules. More importantly, from the front views, the optimized chromophore D molecular structure was close to spherical, which can reduce the intermolecular dipole–dipole interaction and favor the movement of the chromophore during polarization.

### 2.5. Electric Field Poling and EO Property Measurements

In order to study the relationship between macroscopic and microscopic properties of organic electro-optical polymer materials, polymer films doped with 15 wt%, 25 wt%, and 35 wt% chromophores in amorphous polycarbonate (APC) were prepared. Then, the obtained solutions were filtered through a 0.22-μm PTEE filter and spin-coated onto indium tin oxide (ITO) glass substrates. The contact poling process was carried out at a temperature above the glass transition temperature (T_g_) of the polymer, and the poling temperature is shown in Table S1. In order to reduce the effect of multiple reflections, we selected ITO thin-bottom electrodes with low reflectivity and good transparency. The golden film by sputter has good antioxidant properties and conductivity, and it was chosen as the top electrode for poling and the perfect reflection. The polymer was sandwiched between two parallel electrodes, and a voltage with a typical 100 V/μm under the conventional contact poling condition was applied to the polymer. The polarized film was measured for the $r_{33}$ value at 1310 nm wavelength using the Teng-Man simple reflection method. The $r_{33}$ values were calculated with the following equation [34]:

r33=3λIm4πVmIc(n2−sin2θ)32(n2−2sin2θ)1sin2θ

where $r_{33}$ is the EO coefficient of the poled polymer; *λ* is the optical wavelength; *θ* is the incidence angle; *I_c_* is the output beam intensity; *I_m_* is the amplitude of the modulation; *V_m_* is the modulating voltage; and *n* is the refractive indices of the polymer films.

The measured $r_{33}$ values depend on the chromophore number density (N), hyperpolarizability (β), and poling efficiency, described by the $\left\langle \cos^{3}\theta \right\rangle$ order parameter, as indicated by Ref. [35].

$$r_{33}=|2Nf\left(\omega \right)\beta \langle \cos^{3}\theta \rangle /n^{4}|$$

where n is the refractive index of the film, and the f(ω) term describes the electric field (Debye–Onsager) factors, which remain relatively constant for the associated chromophores under similar loading densities. The $\cos^{3}\theta$ term is the acentric order parameter. θ is the angle between the permanent dipole moment of the chromophores and the applied electric field.

The electro-optical coefficient ($r_{33}$) should increase linearly with chromophore density, dipole moment, first hyperpolarizability, and the strength of the electric field for poling, when the intermolecular electrostatic interaction is neglected. However, these chromophores with larger dipole moments actually generate an intermolecular electrostatic field dipole–dipole interaction that leads to antiparallel stacking of chromophores. The r_33_ value of films A/APC, B/APC, C/APC, and D/APC with different concentration are shown in Table 4. For chromophore A, the $r_{33}$ values gradually improved from 13 pm/V (15 wt%) to 18 pm/V (25 wt%), but they decreased to 15 pm/V (35 wt%). For chromophore B, the $r_{33}$ values increased from 17 pm/V (15 wt%) to 28 pm/V (25 wt%) and also decreased to 18 pm/V (35 wt%). However, the $r_{33}$ values of chromophore C increased from 23 pm/V (15 wt%), 32 pm/V (25 wt%) to 38 pm/V (35 wt%). A similar trend of improvement was also observed for chromophore D, whose $r_{33}$ values gradually improved from 29 pm/V (15 wt%), 43 pm/V (25 wt%) to 54 pm/V (35 wt%). When the concentration of chromophore is low (15 wt% and 25 wt%), the $r_{33}$ values of all chromophores increased with the increase of concentration. As the chromophore loading increased to 35 wt%, the intermolecular electrostatic interaction cannot be ignored. The $r_{33}$ values of the chromophores A and B without hindrance groups showed a downward trend, but the r_33_ values of chromophores C and D with hindrance groups were higher than the films with 25 wt% content. It indicated that the *3,5*-bis(trifluoromethyl)benzene hindrance group plays the role of an isolating group well.

The chromophores B and D showed the larger $r_{33}$ than the relative chromophores A and C, because of the stronger electron-withdrawing ability of CF_3_-Ph-TCF compared to TCF. For chromophore D, the largest $r_{33}$ value is attributed to both the CF_3_-Ph-TCF acceptor and the large hindrance group. With the CF_3_-Ph-TCF electron acceptor, the EO coefficient increased obviously, because the first hyperpolarizability (β) of chromophore D increased. In addition, because the flexible hindrance group occupied some steric hindrance, which weakened the dipole–dipole interaction during the poling process, the electro-optic activity of chromophore D was greatly enhanced, which proved that the isolating group was very effective in weakening the dipole-dipole interaction.

## 3. Experiments

### 3.1. Materials

All chemicals are commercially available and are used without further purification unless otherwise stated. *N,N*-dimethylformamide (DMF), tetrahydrofuran (THF), and ether were distilled over calcium hydride and stored over molecular sieves (pore size 3 Å). TLC analyses were carried out on 0.25 mm-thick precoated silica plates, and spots were visualized under UV light. Chromatography on silica gel was carried out on a Kieselgel (200–300 mesh).

### 3.2. Measurements and Instrumentation

^1^H NMR, ^13^C NMR, ^19^F NMR spectra were recorded on an Advance Bruker 400 M (400 MHz) NMR spectrometer (tetramethylsilane as the internal reference, Bruker, Zurich/Ferrandon, Switzerland), and Fourier transform infrared (FTIR) spectra were recorded using a Varian 3100 FT-IR spectrometer (Varian, Melbourne, Australia) as shown in Figures S3–S10. Electrospray ionization (ESI) mass spectra were recorded on an AB SCIEX TripleTOF 4600 mass spectrometer (AB SCIEX, Foster City, CA, USA). The UV–Vis spectra were performed on a Cary 5000 photo spectrometer (Agilent, Palo Alto, CA, USA). The TGA was determined by using a TA5000-2950TGA (TA Co., Delaware, OH, USA) with a heating rate of 10 °C·min^−1^ under the protection of nitrogen. The T_g_ or melt points were determined by TA DSC Q10 with a heating rate of 10 °C·min^−1^ under the protection of nitrogen. The DFT calculations using Gaussian 09 were carried out at the hybrid B3LYP level by employing the split valence 6-31+G* basis set [35]. The Au electrode was deposited by LJUHV-SP3 magnetic sputter. The refractive index of the polymer film was measured using Metricon Prism Coupler Model 2010/M (Metricon, Piscataway, NJ, USA).

### 3.3. Synthesis of Compound

8-(6-chlorohexyl)oxy)-1,1,7,7-tetramethyljulolidine-9-carboxaldehyde (compound **4**) and (compound **5**) were synthesized according to our previous work [36]. 1-(Azidomethyl)-3,5-bis-(trifluoromethyl)benzene (compound **7**) was prepared according to the literature [37]. TCF and CF_3_-TCF acceptors were synthesized according to the literature [38].

### 3.4. Synthesis of Compound **2**

A solution of compound **1** (1.62 g, 10 mmol) and tributyl phosphonium bromide (PPh_3_·HBr) (3.43 g, 10 mmol) in a lot of chloroform was refluxed for 5 h, then cooled to room temperature. After the removal of the solvent, the residue was dissolved using a small amount of chloroform, and it was dropped into a large amount of ether and was filtered to obtain white sediment in 85% yield (4.1 g). ESI–MS (C_28_H_24_BrOP): calcd: 486.07; found: 486.22.

### 3.5. Synthesis of Compound **6**

To a solution of compound **5** (6.4 g, 12.3 mmol) in anhydrous chloroform (50 mL) at ambient temperature was successively added anhydrous DMF (1.0 mL, 12.3 mmol) and POCl_3_ (1.7 mL, 18.5 mmol), and the mixture was refluxed for one night. After hydrolysis for 2 h under vigorous stirring at ambient temperature using an aqueous solution of sodium acetate 2 M (400 mL), the product was extracted using CH_2_Cl_2_. When the solvent was removed, the crude product was purified by chromatography to receive a product (56% yield, 3.77 g). IR (KBr), *v*_max_·cm^−1^: 3456 (Intramolecular hydrogen bond, O-H), 3268 (Ph-H, C=C-H), 2937, 2866 (C-H), 1666 (-CHO), 1582, 1511,1433 (aromatic ring), 1265 (Ph-O-C), 1089 (C-Cl). HRMS (ESI) (M+H)^+^: calcd for (C_34_H_42_ClNO_3_+H)^+^: 548.2926; found: 548.2917.

^1^H NMR (400 MHz, CDCl_3_) δ 9.57 (s, 1H), 7.42 (s, 1H), 7.14 (d, *J* = 8.5 Hz, 2H), 6.96 (d, *J* = 8.5 Hz, 2H), 6.68 (s, 1H), 4.62 (d, *J* = 2.2 Hz, 2H), 3.88 (t, *J* = 6.5 Hz, 2H), 3.50 (t, *J* = 6.6 Hz, 2H), 3.07 (m, 4H), 2.42 (s, 1H), 1.89–1.75 (m, 4H), 1.67–1.60 (m, 2H), 1.50 (m, 6H), 1.34 (s, 6H), 0.75 (s, 6H).

^13^C NMR (101 MHz, CDCl_3_) δ 193.79, 158.84, 157.14, 147.96, 145.28, 135.65, 131.04, 128.26, 127.22, 125.69, 121.67, 115.51, 114.64, 78.73, 76.45, 75.55, 55.95, 47.42, 46.84, 45.06, 39.84, 36.03, 32.64, 31.81, 30.39, 30.09, 27.01, 25.69.

### 3.6. Synthesis of Chromophore A

A solution of compound **6** (0.55 g, 1 mmol) and TCF acceptor (0.32 g, 1.6 mmol) in ethanol (20 mL) was allowed to stir at 70 °C for 3 h, and after removal of the solvent under reduced pressure, the crude product was additionally purified by silica chromatography to obtain A as a green solid in 56% yield (0.04 g). IR (KBr), *v*_max_·cm^−1^: 3437 (Intramolecular hydrogen bond, O-H), 3288 (Ph-H, C=C-H), 2931, 2866 (C-H), 2218–2211 (C≡N, C≡C), 1614, 1556, 1511 (aromatic ring), 1310 (Ph-O-C), 1082 (C-Cl). HRMS (ESI) (M+H)^+^: calcd for (C_45_H_49_ClN_4_O_3_+H)^+^: 729.3566; found: 729.3545.

^1^H NMR (400 MHz, CDCl_3_) δ 7.97 (d, *J* = 15.1 Hz, 1H), 7.31 (s, 1H), 7.12–7.03 (m, 4H), 6.47 (s, 1H), 5.69 (d, *J* = 15.1 Hz, 1H), 4.68 (d, *J* = 2.0 Hz, 2H), 3.85 (t, *J* = 6.5 Hz, 2H), 3.52 (t, *J* = 6.5 Hz, 2H), 3.15 (m, 4H), 2.46 (s, 1H), 1.83 (m, 4H), 1.69–1.61 (m, 2H), 1.58–1.48 (m, 12H), 1.33 (s, 6H), 0.73 (s, 6H).

^13^C NMR (101 MHz, CDCl_3_) δ 176.70, 173.02, 160.20, 157.47, 154.76, 146.53, 144.74, 134.61, 130.68, 129.73, 127.33, 126.50, 122.06, 116.35, 116.26, 113.12, 112.21, 112.07, 110.97, 96.48, 91.83, 78.34, 75.82, 55.90, 53.57, 47.63, 47.02, 45.11, 39.25, 35.43, 32.51, 32.44, 31.68, 30.91, 29.99, 29.77, 29.66, 26.80, 26.40, 25.59, 24.40.

### 3.7. Synthesis of Chromophore B

A solution of compound **6** (0.55 g, 1 mmol) and CF_3_-Ph-TCF acceptor (0.45 g, 1 mmol) in ethanol (20 mL) was allowed to stir at 70 °C for 3 h, and after removal of the solvent under reduced pressure, the crude product was additionally purified by silica chromatography to obtain B as a green solid in 70% yield (0.59 g). IR (KBr), *v*_max_·cm^−1^: 3450 (Intramolecular hydrogen bond, O-H), 3275 (Ph-H, C=C-H), 2937, 2859 (C-H), 2224–2211 (C≡N, C≡C), 1608, 1524, 1491 (aromatic ring), 1310 (Ph-O-C), 1089 (C-Cl). HRMS (ESI) (M+H)^+^: calcd for (C_50_H_48_ClF_3_N_4_O_3_+H)^+^: 845.3440; found: 845.3408.

^1^H NMR (400 MHz, CDCl_3_) δ 7.44–7.33 (m, 3H), 7.30 (d, *J* = 6.2 Hz, 3H), 7.19 (s, 1H), 7.02 (s, 4H), 6.54 (s, 1H), 5.82 (d, *J* = 14.4 Hz, 1H), 4.68 (s, 2H), 3.80 (t, *J* = 6.3 Hz, 2H), 3.52 (t, *J* = 6.4 Hz, 2H), 3.22 (d, *J* = 4.9 Hz, 4H), 2.45 (s, 1H), 1.79 (d, *J* = 5.8 Hz, 4H), 1.63 (s, 2H), 1.50 (s, 6H), 1.31 (s, 6H), 0.72 (s, 6H).

^13^C NMR (101 MHz, CDCl_3_) δ 176.40, 161.38, 160.45, 157.46, 156.00, 148.28, 147.54, 135.77, 130.79, 130.76, 130.60, 129.60, 129.31, 128.00, 127.33, 126.34, 123.63, 122.56, 120.78, 117.29, 116.27, 112.40, 112.30, 111.73, 111.59, 78.26, 77.67, 75.79, 55.90, 54.87, 54.51, 48.02, 47.35, 45.15, 38.82, 35.00, 32.45, 32.39, 31.63, 29.90, 29.39, 29.36, 29.33, 26.77, 25.65.

### 3.8. Synthesis of Compound **8**

Compound **6** (0.66 g, 1.1 mmol), compound **7** (0.27 g, 1 mmol), CuSO_4_·5H_2_O (10 mol%), NaHCO_3_ (20 mol%), and ascorbic acid (20 mol%) were dissolved in tert-butanol/H_2_O (10 mL/10 mL) under nitrogen in a Schlenk flask. The mixture was stirred at 25 °C overnight, then extracted with chloroform and washed with 1 N HCl, 1 N NH_4_OH and water subsequently. The organic layer was dried over MgSO_4_. After filtration and removal of the solvent under vacuum, the crude product was purified using silica chromatography and eluted with acetone: petroleum ether (1:4) to renderw compound **8** as a red solid in 85% yield (0.69 g). IR (KBr), *v*_max_·cm^−1^: 3444 (Intramolecular hydrogen bond, O-H), 3152 (Ph-H, C=C-H), 2937, 2859 (C-H), 1673 (-CHO), 1585, 1511, 1459 (aromatic ring), 1283 (Ph-O-C), 1174 (N=N), 1082 (C-Cl). HRMS (ESI) (M+H)^+^: calcd for (C_43_H_47_ClF_6_N_4_O_3_+H)^+^: 817.3314; found: 817.3302.

^1^H NMR (400 MHz, CDCl_3_) δ 9.67 (s, 1H), 7.89 (s, 1H), 7.76 (s, 2H), 7.65 (s, 1H), 7.48 (s, 1H), 7.19 (d, *J* = 8.7 Hz, 2H), 7.02 (d, *J* = 8.7 Hz, 2H), 6.77 (s, 1H), 5.67 (s, 2H), 5.55–5.50 (m, 1H), 5.23 (s, 2H), 3.96 (d, *J* = 6.6 Hz, 2H), 3.57 (t, *J* = 6.6 Hz, 2H), 3.17 (t, *J* = 9.3 Hz, 3H), 1.88 (m, 6H), 1.61 (m, 6H), 1.43 (s, 6H), 0.81 (s, 6H).

^13^C NMR (101 MHz, CDCl_3_) δ 193.82, 158.74, 157.62, 148.02, 145.41, 145.38, 137.10, 133.01, 132.78, 132.56, 132.34, 131.12, 128.17, 127.97, 127.15, 123.77, 122.94, 122.92, 122.85, 122.82, 121.96, 115.25, 76.38, 62.25, 53.03, 47.39, 46.83, 45.02, 39.67, 35.84, 32.58, 32.53, 31.72, 30.36, 30.34, 30.26, 30.09, 29.98, 29.75, 29.68, 26.93, 25.62.

^19^F NMR (565 MHz, CDCl_3_) δ −62.89 (s, CF_3_).

### 3.9. Synthesis of Chromophore C

A solution of compound **8** (0.82 g, 1 mmol) and TCF acceptor (0.32 g, 1.6 mmol) in ethanol (20 mL) was allowed to stir at 70 °C for 3 h, and after removal of the solvent under reduced pressure, the crude product was additionally purified by silica chromatography to obtain C as a green solid in 52% yield (0.52 g). IR (KBr), *v*_max_·cm^−1^: 3456 (Intramolecular hydrogen bond, O-H), 3199 (Ph-H, C=C-H), 2923, 2844 (C-H), 2225 (C≡N, C≡C), 1514, 1478 (aromatic ring), 1367 (Ph-O-C), 1183 (N=N), 1110 (C-Cl). HRMS (ESI) (M+H)^+^: calcd for (C_54_H_54_ClF_6_N_7_O_3_+H)^+^: 998.3954; found: 998.3937.

^1^H NMR (400 MHz, CDCl_3_) δ 7.86 (s, 1H), 7.77 (d, *J* = 13.0 Hz, 3H), 7.68 (s, 1H), 7.36 (s, 1H), 7.27 (s, 1H), 7.15–7.10 (m, 4H), 6.62 (s, 1H), 5.79 (d, *J* = 15.1 Hz, 1H), 5.70 (s, 2H), 5.32 (s, 2H), 3.92 (t, *J* = 6.6 Hz, 2H), 3.59 (t, *J* = 6.6 Hz, 2H), 3.44–3.15 (m, 4H), 1.99–1.91 (m, 2H), 1.86 (m, 2H), 1.75–1.52 (m, 14H), 1.48–1.37 (m, 6H), 0.91–0.73 (m, 6H).

^13^C NMR (101 MHz, CDCl_3_) δ 176.31, 173.22, 160.08, 157.88, 154.20, 146.43, 144.81, 144.18, 137.47, 134.74, 132.83, 132.60, 132.38, 132.16, 130.83, 129.70, 128.17, 127.37, 126.55, 123.82, 123.02, 122.75, 122.72, 122.70, 122.10, 122.01, 116.55, 116.28, 112.99, 112.19, 111.76, 111.66, 96.40, 62.34, 62.13, 53.75, 52.87, 47.63, 47.01, 45.04, 39.19, 35.31, 32.51, 32.45, 31.66, 30.03, 29.64, 26.81, 26.68, 25.65.

^19^F NMR (565 MHz, CDCl_3_) δ −62.84 (s, CF_3_).

### 3.10. Synthesis of Chromophore D

A solution of compound **8** (0.82 g, 1 mmol) and CF_3_-Ph-TCF acceptor (0.45 g, 1 mmol) in ethanol (20 mL) was allowed to stir at 70 °C for 3 h, and after removal of the solvent under reduced pressure, the crude product was additionally purified by silica chromatography to obtain D as a green solid in 67% yield (0.74 g). IR (KBr), *v*_max_·cm^−1^: 3437 (Intramolecular hydrogen bond, O-H), 2931, 2854 (C-H), 2231 (C≡N), 1569, 1439 (aromatic ring), 1310 (Ph-O-C), 1187 (N=N), 1109 (C-Cl). HRMS (ESI) (M+H)^+^: calcd for (C_59_H_53_ClF_9_N_7_O_3_+H)^+^: 1114.3827; found: 1114.3811.

^1^H NMR (400 MHz, CDCl_3_) δ 7.87 (s, 1H), 7.75 (s, 2H), 7.67 (d, *J* = 8.1 Hz, 1H), 7.50 (dd, *J* = 8.1, 5.0 Hz, 1H), 7.48–7.43 (m, 2H), 7.41 (d, *J* = 7.8 Hz, 2H), 7.26 (s, 2H), 7.08 (q, *J* = 8.6 Hz, 4H), 6.64 (s, 1H), 5.92 (d, *J* = 11.9 Hz, 1H), 5.68 (s, 2H), 5.31 (s, 2H), 3.84 (t, *J* = 6.6 Hz, 2H), 3.59 (t, *J* = 6.3 Hz, 2H), 3.38–3.18 (m, 4H), 1.85 (s, 4H), 1.76–1.66 (m, 2H), 1.55 (s, 6H), 1.38 (s, 6H), 0.76 (s, 6H).

^13^C NMR (101 MHz, CDCl_3_) δ 175.99, 161.35, 160.68, 157.98, 156.29, 148.53, 147.46, 144.77, 137.38, 135.77, 132.87, 132.65, 132.42, 132.20, 130.99, 130.79, 130.75, 129.39, 128.17, 127.98, 127.29, 126.71, 125.62, 123.81, 123.22, 123.02, 122.78, 122.44, 122.00, 121.32, 120.19, 117.15, 116.41, 112.30, 111.85, 111.73, 95.30, 95.09, 77.66, 62.32, 60.41, 54.33, 52.91, 47.99, 47.31, 45.10, 38.76, 34.89, 32.42, 32.39, 31.58, 29.91, 29.36, 29.34, 29.20, 29.16, 26.80, 25.49.

^19^F NMR (565 MHz, CDCl_3_) δ −62.87 (s, CF_3_).

## 4. Conclusions

In this work, the novel chromophores A, B, C, and D, based on the tricyanofuran (TCF) and CF_3_-Ph-TCF acceptors, were synthesized and systematically investigated by NMR, MS, and UV–Vis absorption spectroscopy. The energy gap between the ground state and the excited state and the molecular nonlinearity were investigated by UV–Vis absorption spectroscopy and DFT calculations. Theoretical and experimental studies showed that CF_3_-Ph-TCF with super electron-withdrawing ability could greatly improve its microscopic hyperpolarizability (β) and macroscopic EO property compared to conventional TCF acceptors. The macroscopic and microscopic properties of chromophores, including thermal stability, photo-physics, and EO performance, were investigated by experiments and calculations. The different compositions of the chromophores led to the differences in spatial structure, physical properties, and DFT calculation results. Chromophore D with a flexible hindrance group and the CF_3_-Ph-TCF acceptor exhibited the highest EO coefficient of 54 pm/V. The moderate r_33_ values and thermal stability indicate that these new second-order nonlinear optical chromophores have attractive potential applications in devices.

## Figures and Tables

**Table 1:** The thermal properties of the chromophores.

Chromophore	T_d_ (°C)	T_g_ (°C)
Chromophore A	249	98.2
Chromophore B	221	90.4
Chromophore C	243	83.5
Chromophore D	226	80.2

**Table 2:** The maximum UV–Vis–NIR absorption wavelength in different solvents.

Chromophore	λ_max_^a^/ε	λ_max_^b^/ε	λ_max_^c^/ε	λ_max_^d^/ε	λ_max_^e^/ε	λ_max_^f^/ε	Δλ_max_^g^
	(nm)	(nm)	(nm)	(nm)	(nm)	(nm)	(nm)
Chromophore A	666/11.6	702/18.2	674/12.6	625/11.2	685/13.2	693/13.8	77
Chromophore B	739/9.7	764/13.9	764/11.4	730/7.8	774/12.9	772/14.4	44
Chromophore C	670/16.8	702/11.6	671/11.7	625/11.7	683/11.8	690/13.0	77
Chromophore D	742/11.4	765/9.4	766/12.6	728/15.9	774/14.2	772/11.2	46

^a–f^ λ_max_ were measured in toluene, chloroform, tetrahydrofuran, dioxane, acetone, and acetonitrile, respectively. ^g^ Δλ_max_ was difference between λ_max_ ^b^ (or λ_max_ ^e^) and λ_max_ ^d^. ε: molar extinction coefficient, unit: 10^4^ L·mol^−1^·cm^−1^.

**Table 3:** Data from DFT calculations and CV measurements for chromophores.

Chromophores	E_HOMO_/eV	E_LUMO_/eV	ΔE/eV	β_max_/10^−30^ esu	μ (D)
A	−6.10	−2.08	4.01	225	23.8
B	−6.14	−2.19	3.95	241	24.0
C	−6.16	−2.10	4.06	243	20.9
D	−6.19	−2.20	3.99	255	21.3

ΔE = E_LUMO_ − E_HOMO_. Results were calculated from DFT. β values were calculated using Gaussian 09 at the B3LYP/6-31G+*(d,p) level, and the direction of the maximum value is along the charge-transfer axis of the chromophores.

**Table 4:** The $r_{33}$ values of films’ chromophore/APC with different concentrations.

Chromophores	$r_{33}$ with 15 wt%	$r_{33}$ with 25 wt%	$r_{33}$ with 35 wt%
A	13 pm/V	18 pm/V	15 pm/V
B	17 pm/V	28 pm/V	18 pm/V
C	23 pm/V	32 pm/V	38 pm/V
D	29 pm/V	43 pm/V	54 pm/V

## Data Availability

The data presented in this study are available in this article.

## Supplementary Materials

The following supporting information can be downloaded at: https://www.mdpi.com/article/10.3390/molecules28020488/s1, Table S1: The values of glass transition temperature (Tg) and contact poling temperature (Tp) of the chromophore doped APC; Figure S1: The frontier molecular orbitals of chromophores A, B, C and D; Figure S2: The optimized structures of chromophores A, B, C, D; Figure S3: The 1H NMR spectra of the compound 6 and compound 8; Figure S4: The ^1^H NMR spectra of the chromophore A, B, C, and D; Figure S5: The ^13^C NMR spectra of the compound 6 and compound 8; Figure S6: The ^13^C NMR spectra of the chromophore A, B, C, and D; Figure S7: The ^19^F NMR spectrum of the compound 8; Figure S8: The ^19^F NMR spectra of the chromophore C and D; Figure S9: The FTIR spectra of the compound 6 and compound 8; Figure S10: The FTIR spectra of the chromophore A, B, C, and D;
