# Supplementary material for: Systematic Study on Nonlinear Optical Chromophores with Improved Electro-Optic Activity by Introducing 3,5-Bis(trifluoromethyl)benzene Derivative Isolation Groups into the Bridge
Source: Molecules. 2023 Jan 4;28(2):488. doi: 10.3390/molecules28020488 (PMC9862648; doi:10.3390/molecules28020488)
Supplement: Supplementary file 1 [file molecules-28-00488-s001.zip › molecules-2101550-supplementary.pdf]

Supporting Information

Systematic Study on Nonlinear Optical Chromophores with Improved Electro-Optic Activity by Introducing 3,5-Bis(Trifluoromethyl)Benzene Derivative Isolation Groups into the Bridge

Tongtong Liu [1,2], Fuyang Huo [3,*], Changqing Ge [1,2], Ya Li [1,2], Jing He [1,2], Han Zheng [1,2], Qian He [1,2], Yinsen Zhao [1,2], Zhuo Chen[3,*] and Shuhui Bo [1,2*]

1 Optoelectronics Research Centre, School of Science, Minzu University of China, Beijing 100081, China

2 Engineering Research Centre of Photonic Design Software, Ministry of Education, Beijing 100081, China

3 Key laboratory of Bio-inspired Materials and Interfacial Science, Technical Institute of Physics and Chemistry, Chinese Academy of Sciences, Beijing 100190, China

Table S1. The values of glass transition temperature (T_g) and contact poling temperature (T_P) of the chromophore doped APC

Chromophore	A	B	C	D
T_g (15wt%)	154	147	149	148
T_P (15wt%)	155	150	152	149
T_g (25wt%)	135	129	131	133
T_P (25wt%)	137	131	134	135
T_g (35wt%)	130	128	127	129
T_P (35wt%)	134	129	134	133

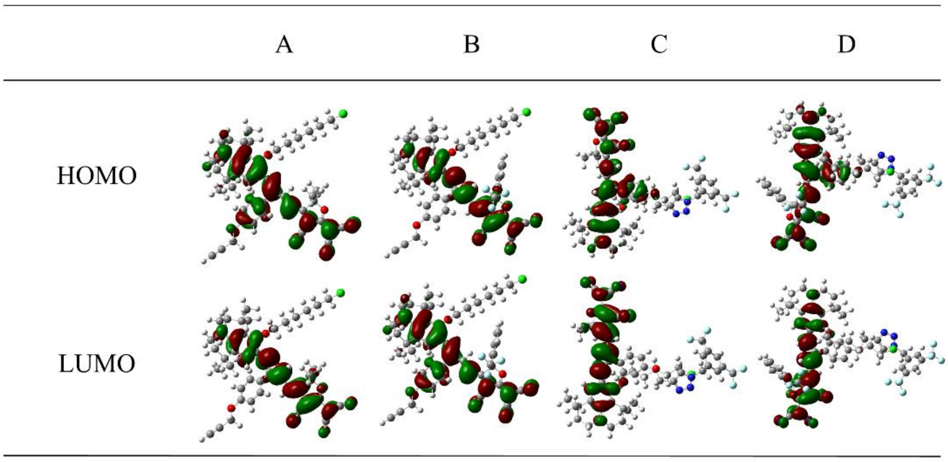

Figure S1. The frontier molecular orbitals of chromophores A, B, C and D.

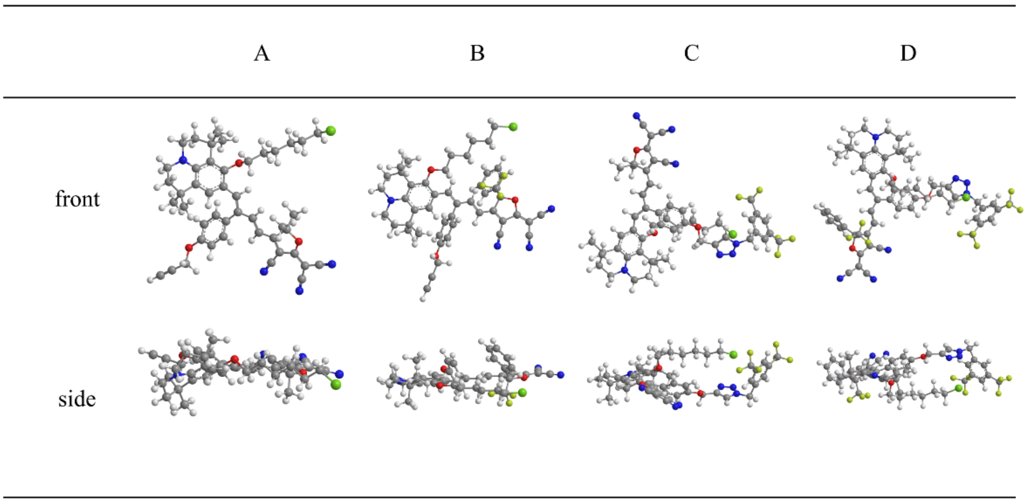

Figure S2. The optimized structures of chromophores A, B, C, D.

Compound 6:

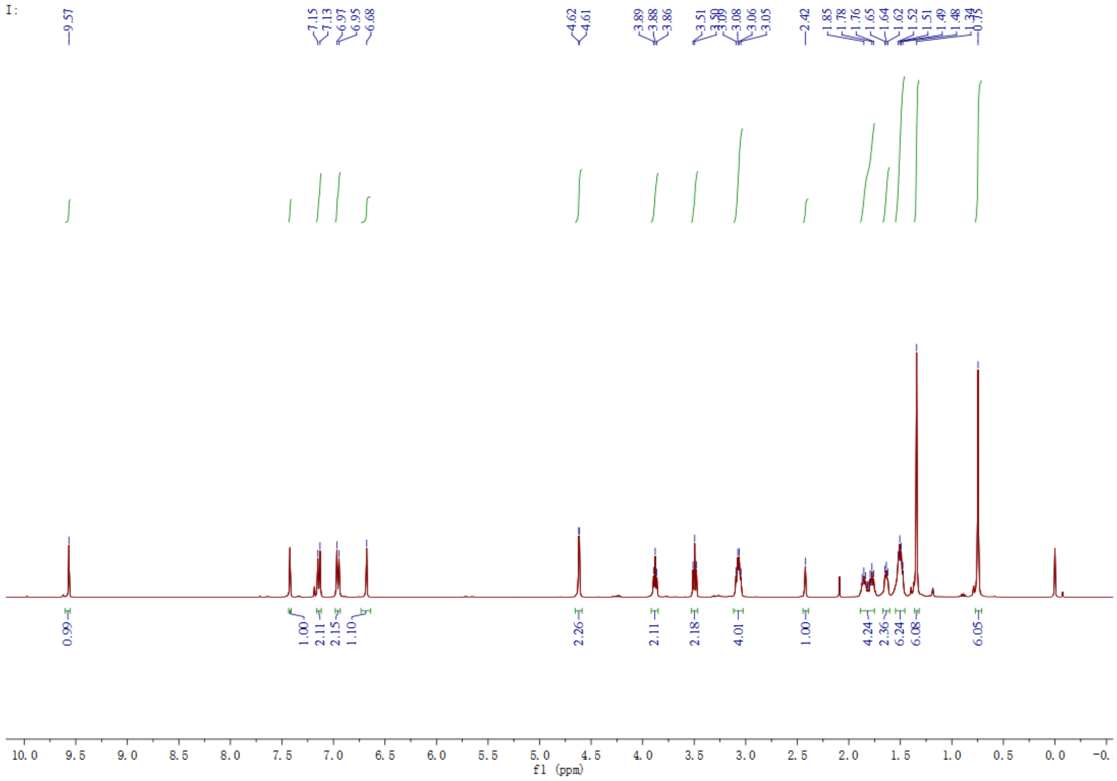

Compound 8:

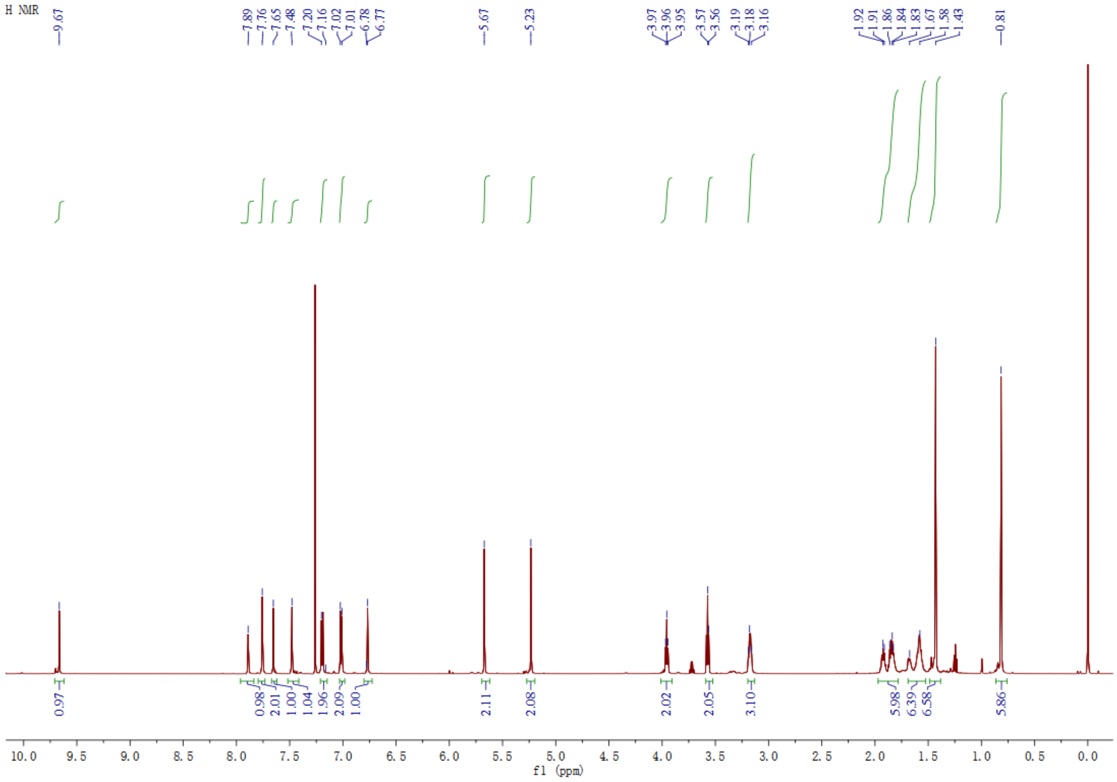

Figure S3. The ^{1}H NMR spectra of the compound 6 and compound 8.

Chromophore A:

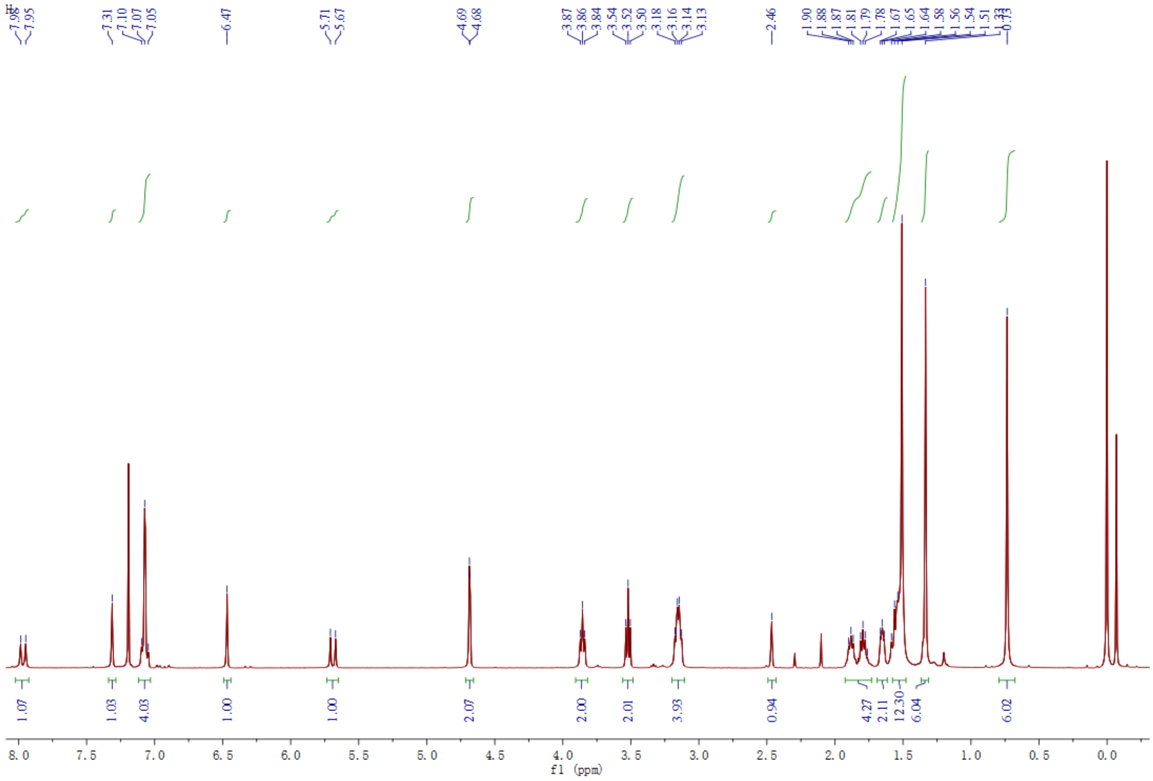

Chromophore B:

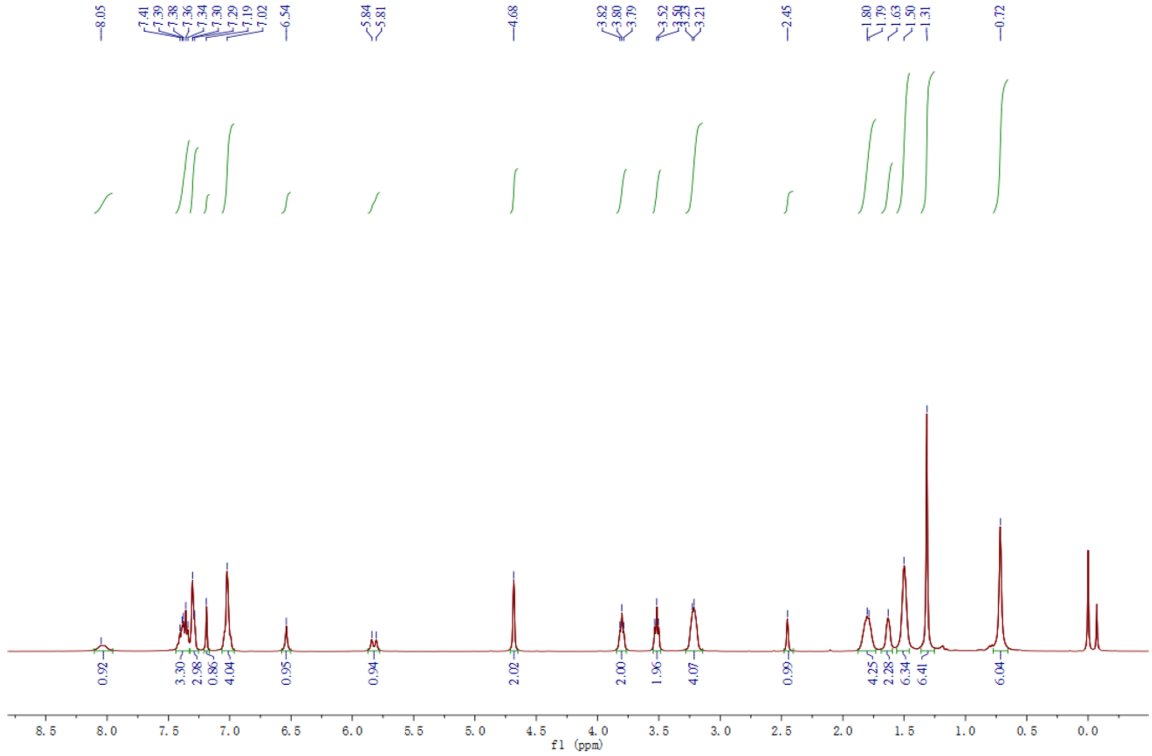

Chromophore C:

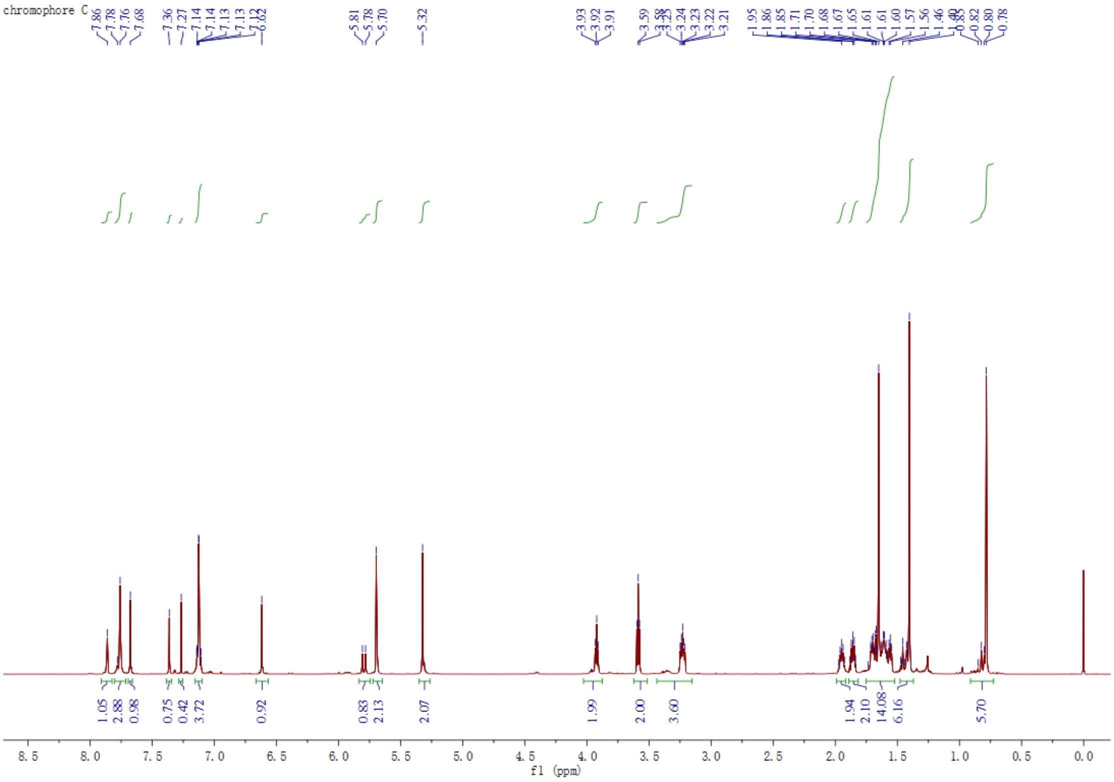

Chromophore D:

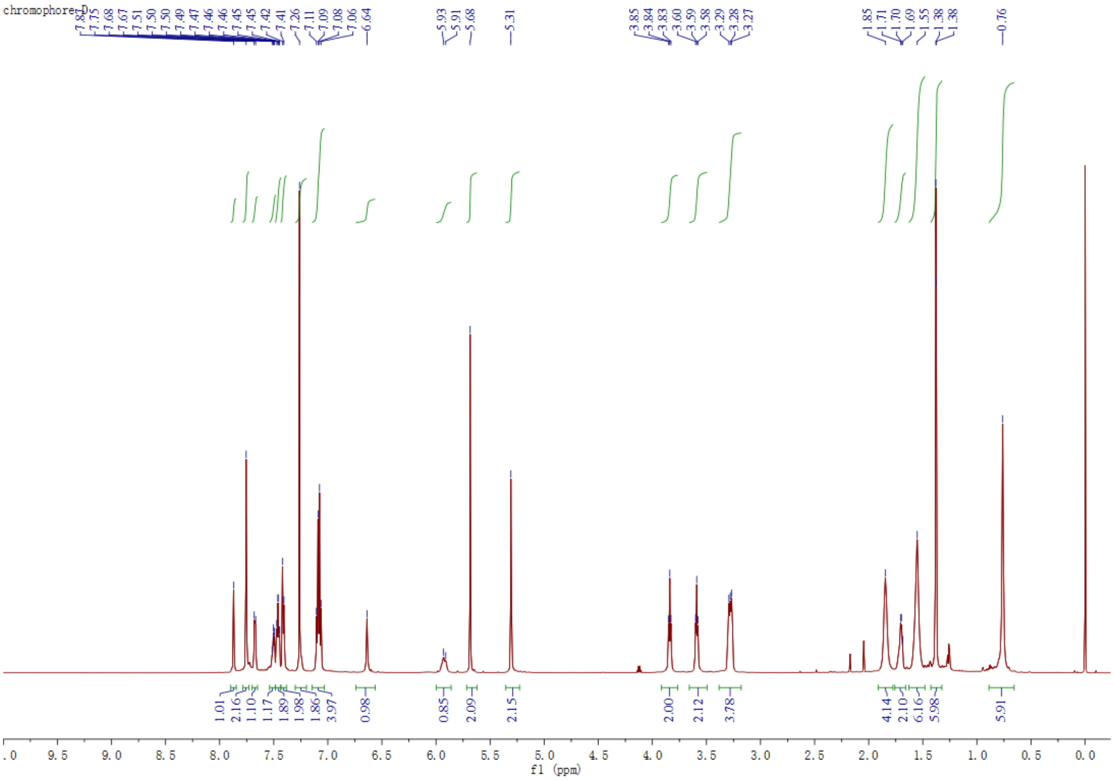

Figure S4. The ^{1}H NMR spectra of the chromophore A, B, C and D.

Compound 6:

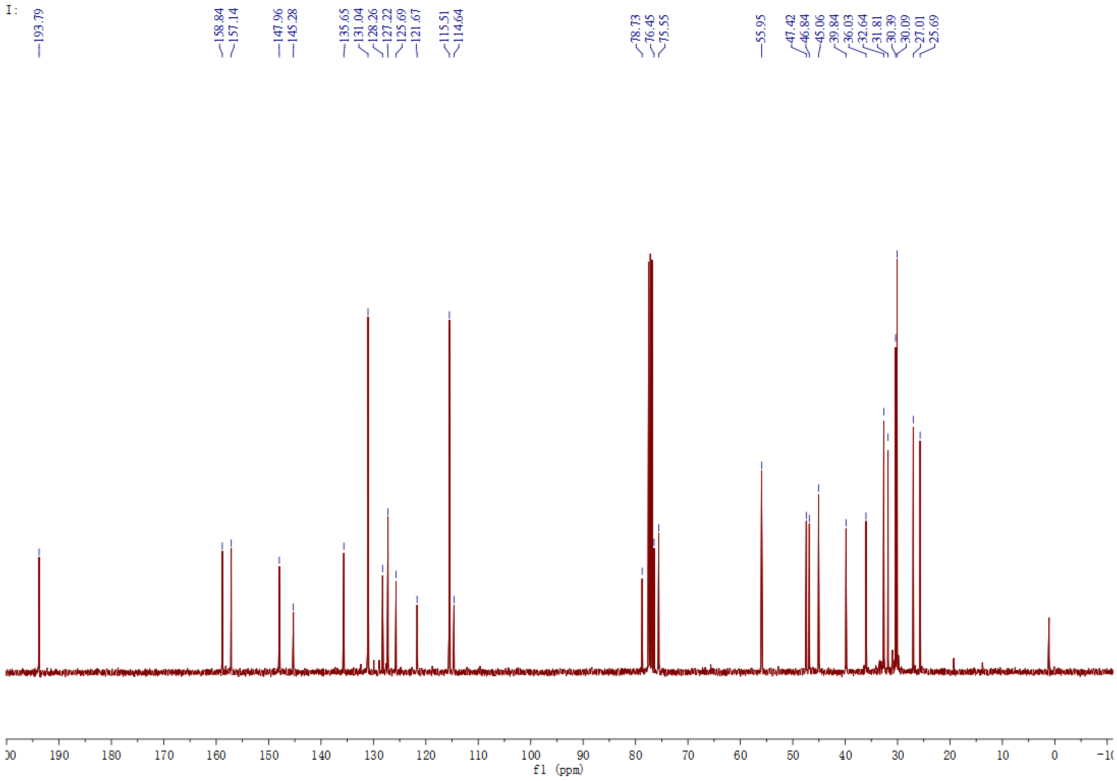

Compound 8:

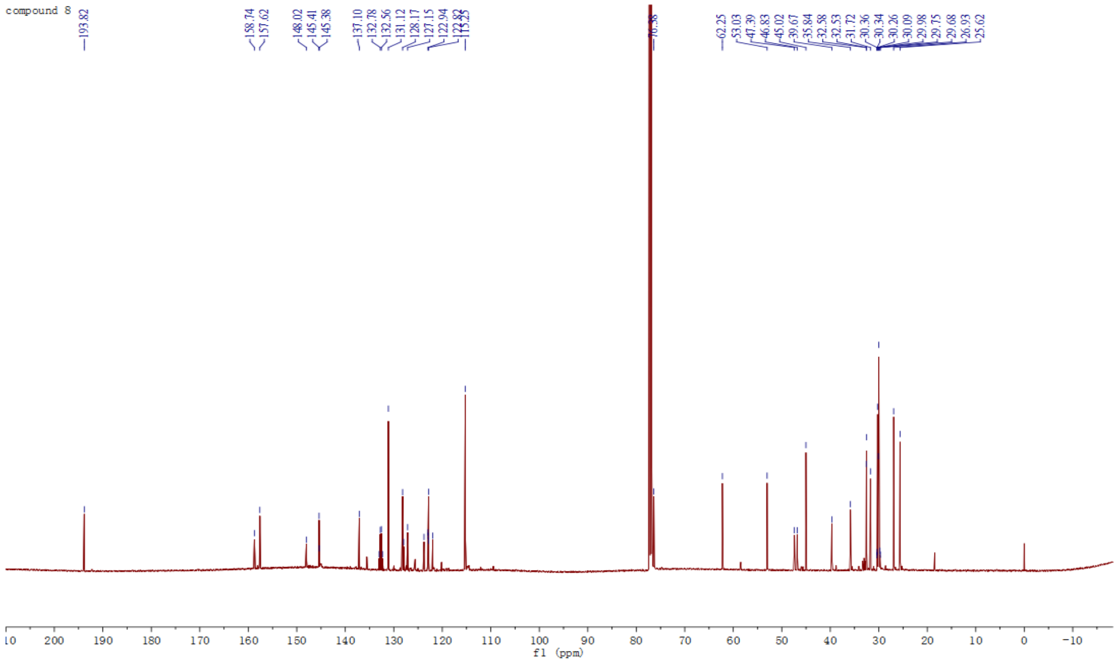

Figure S5. The ^{13}C NMR spectra of the compound 6 and compound 8.

Chromophore A:

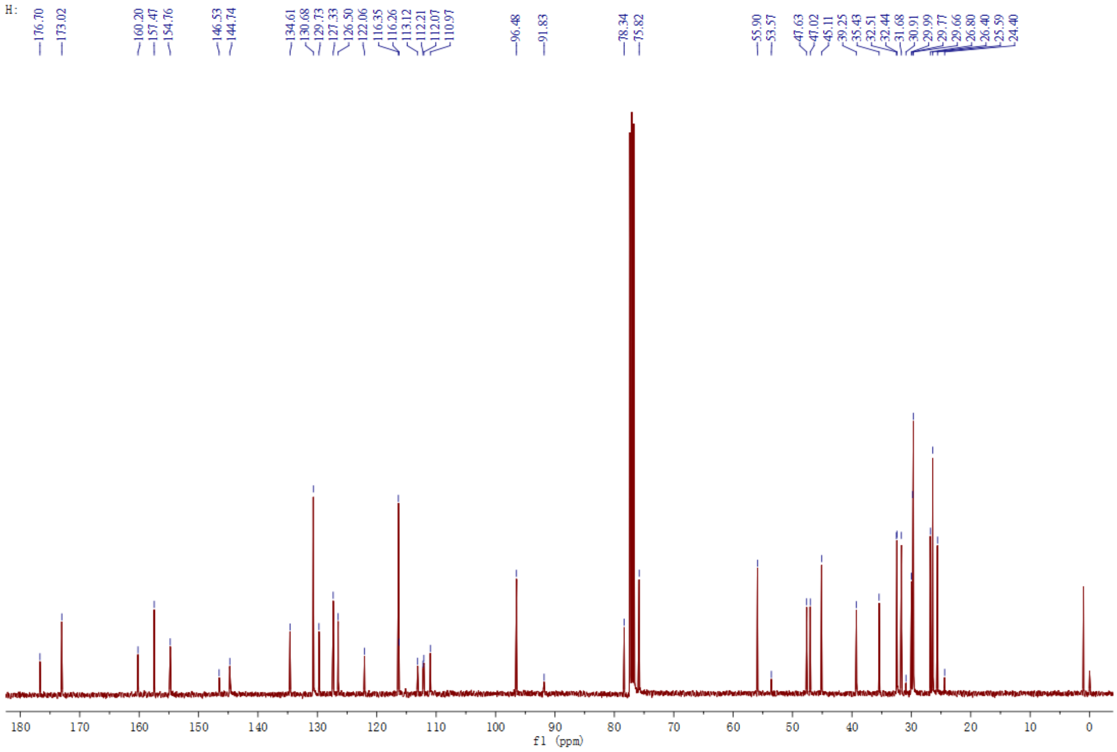

Chromophore B:

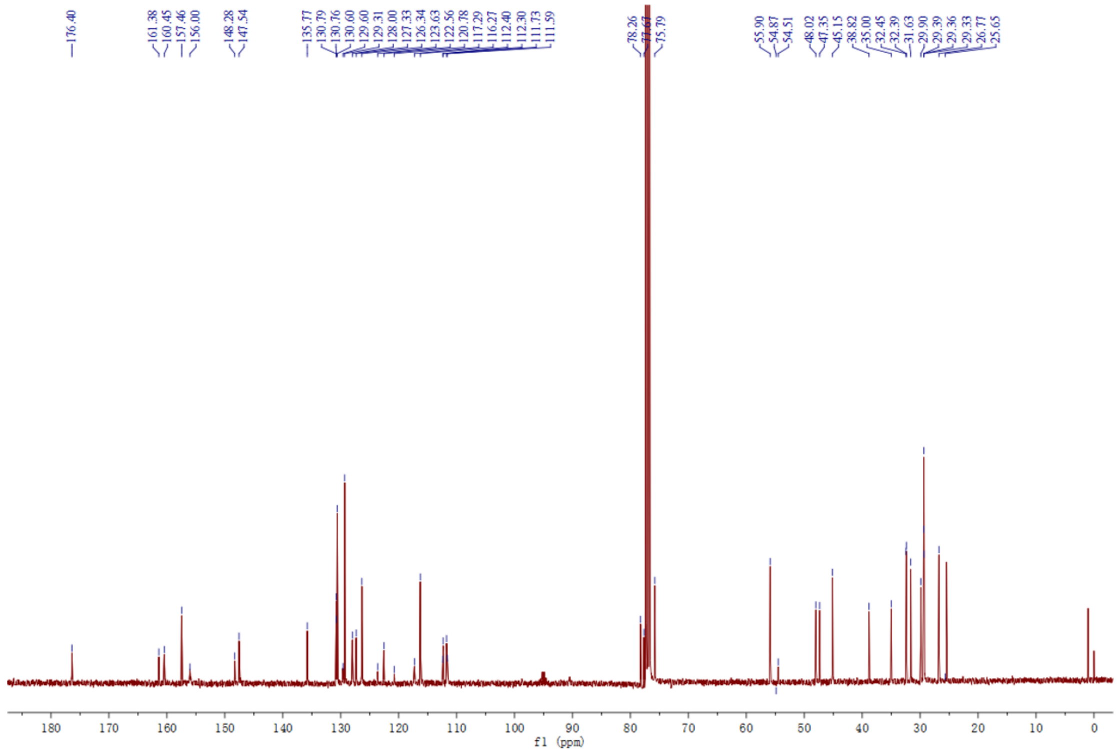

Chromophore C:

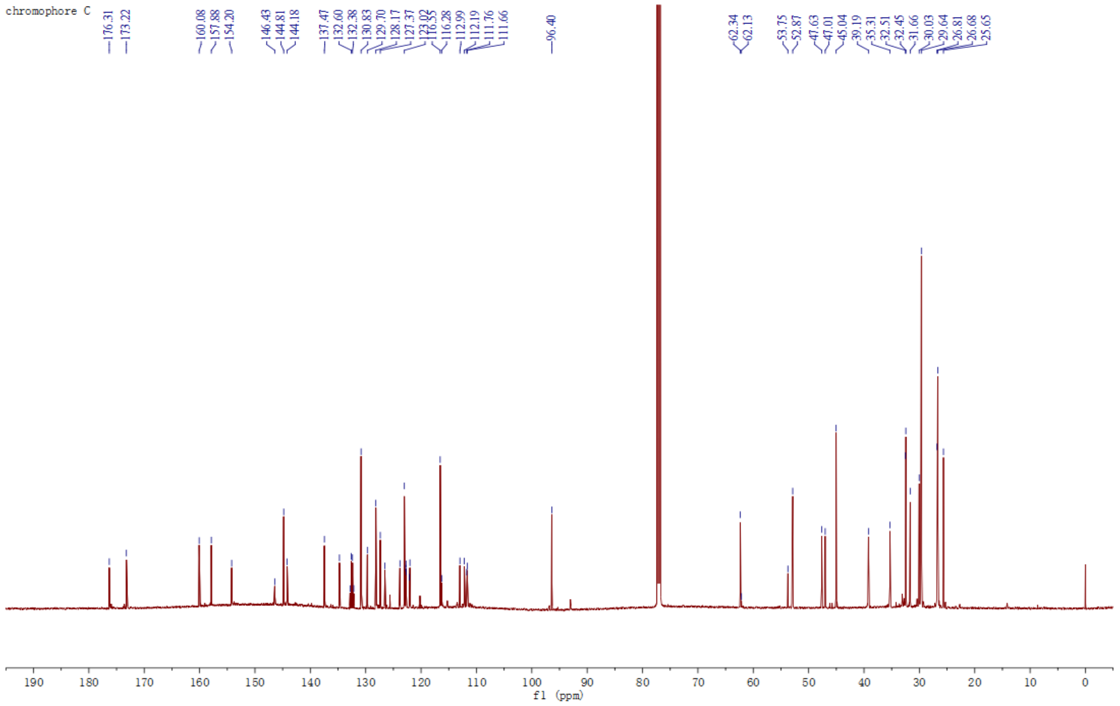

Chromophore D:

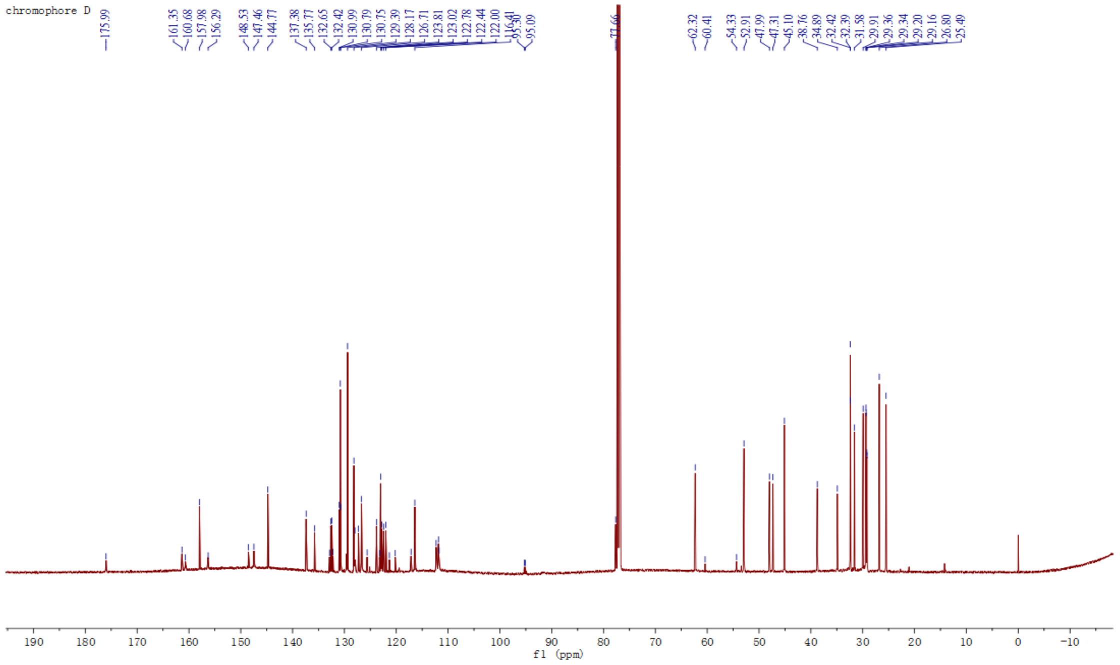

Figure S6. The ^{13}C NMR spectra of the chromophore A, B, C and D.

Compound 8:

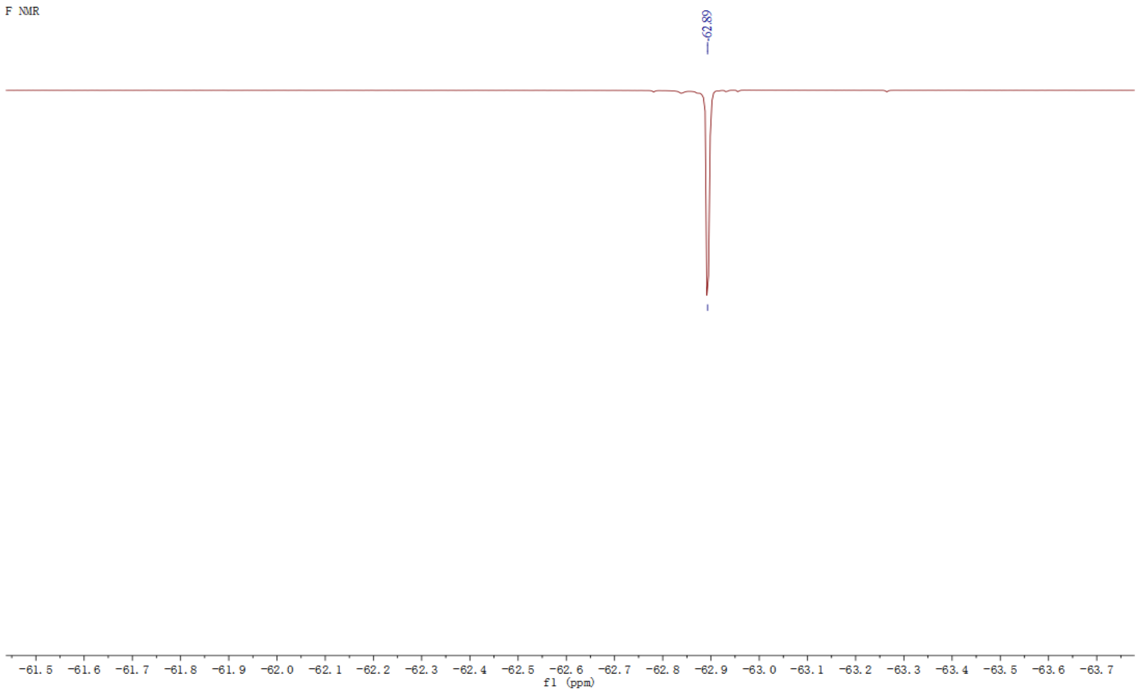

Figure S7. The ^{19}F NMR spectra of the compound 8

Chromophore C:

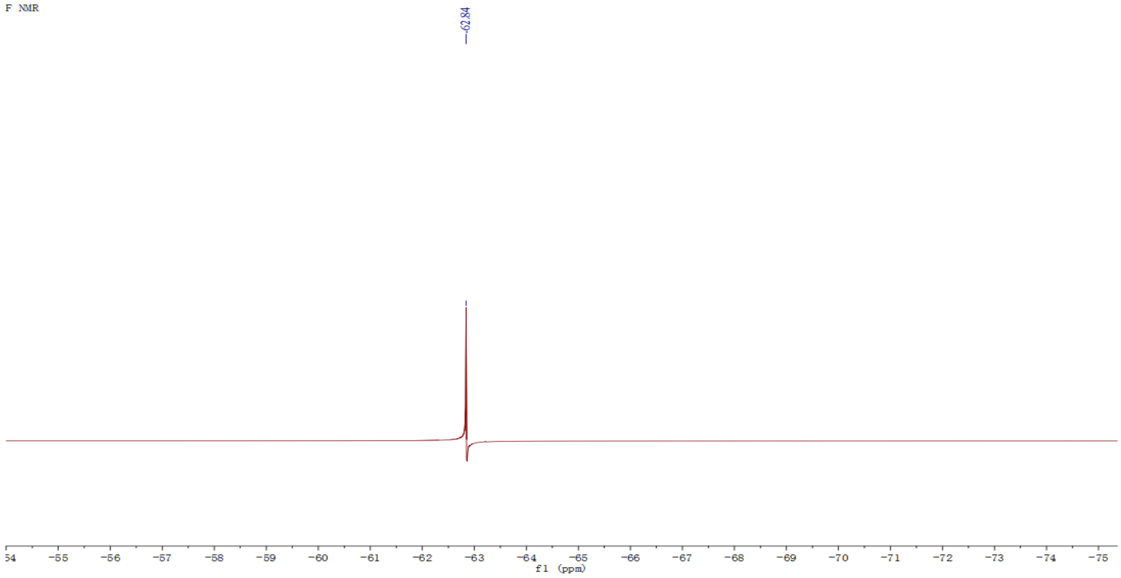

Chromophore D:

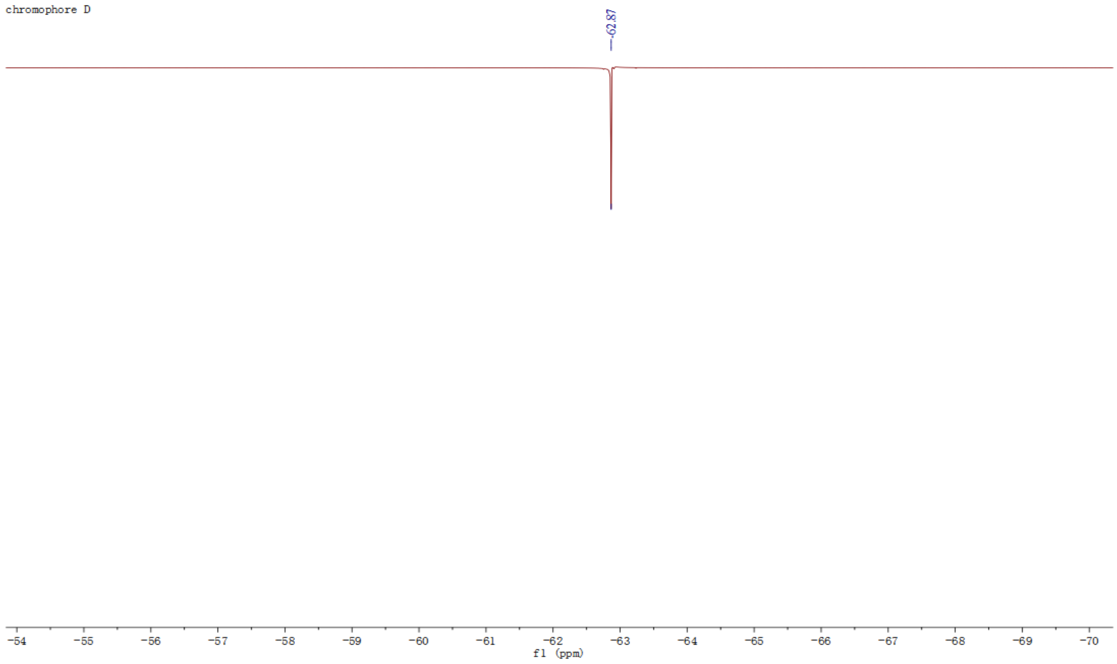

Figure S8. The ^{19}F NMR spectra of the chromophore C and D.

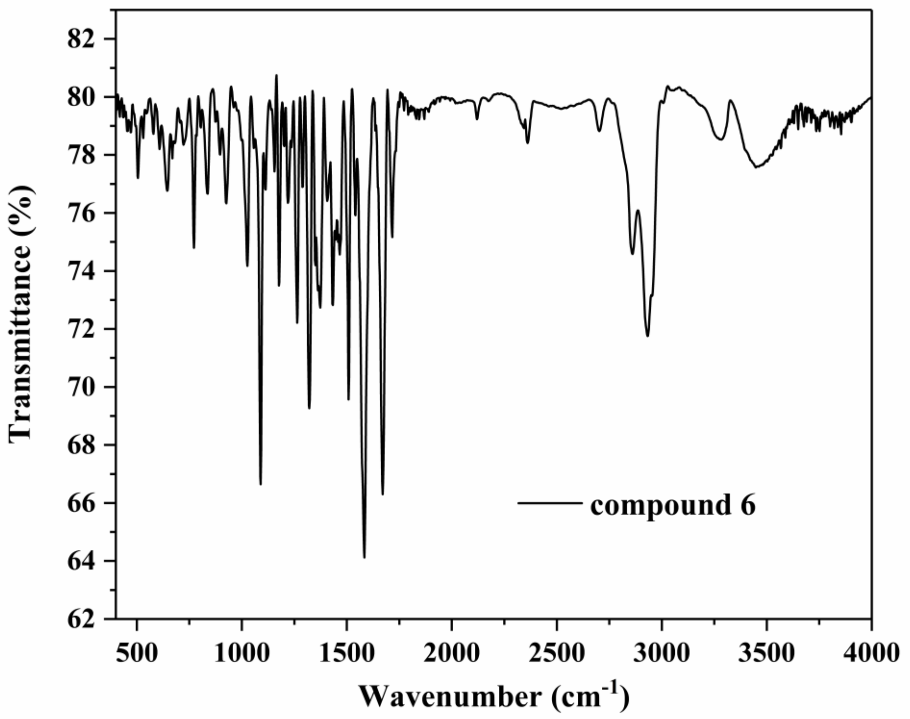

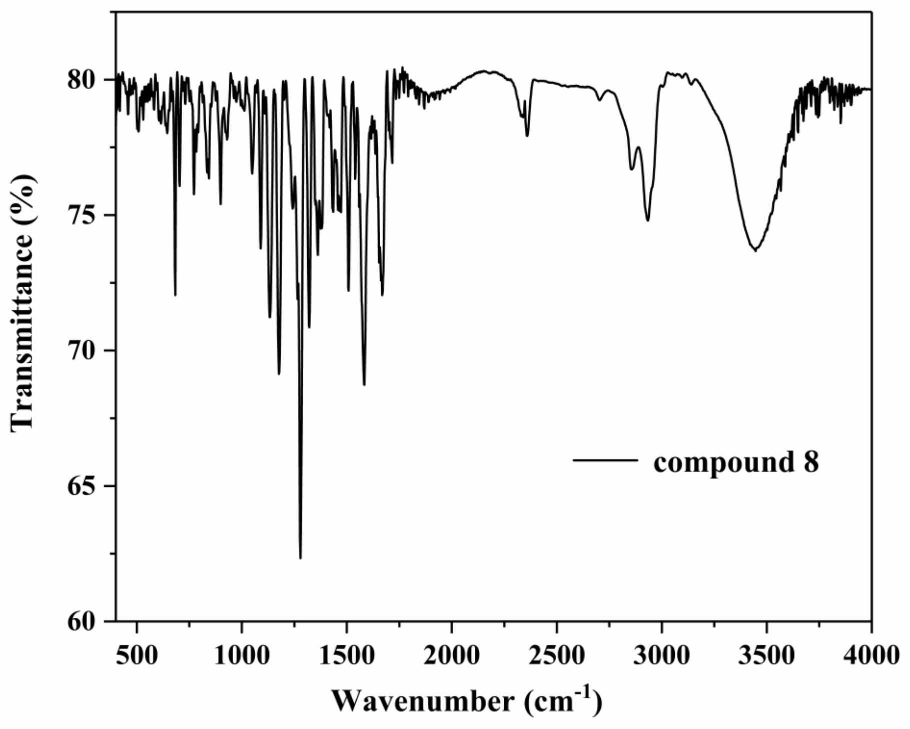

Figure S9. The FTIR spectra of the compound 6 and compound 8.

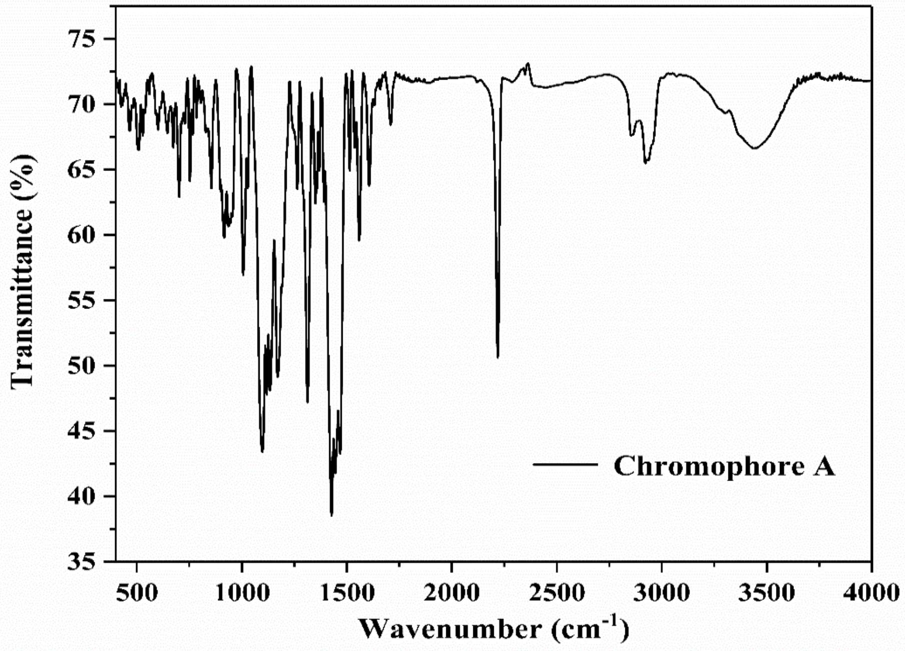
Transmittance (%)
Wavenumber (cm-1)
Chromophore A
75
70
65
60
55
50
45
40
35
500
1000
1500
2000
2500
3000
3500
4000

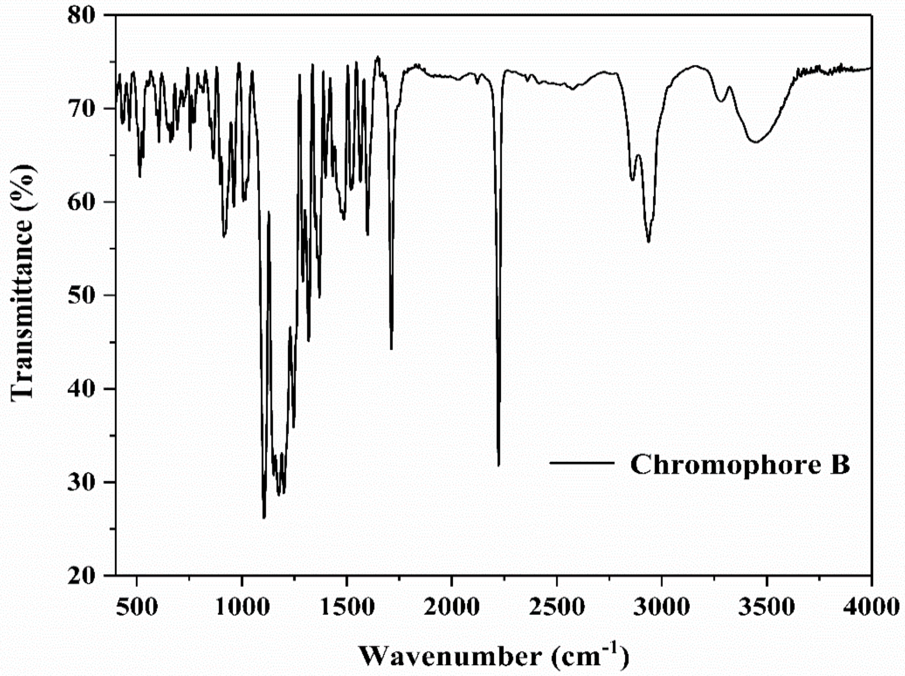
Transmittance (%)
Wavenumber (cm-1)
Chromophore B
80
70
60
50
40
30
20
500
1000
1500
2000
2500
3000
3500
4000

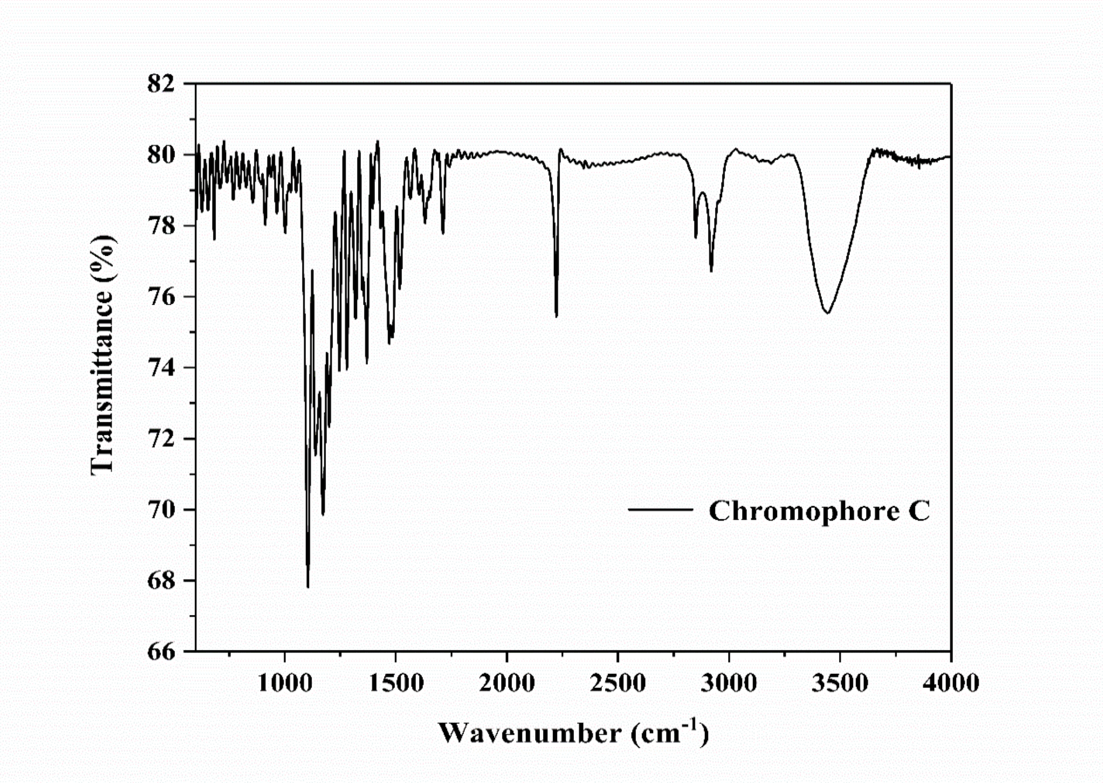

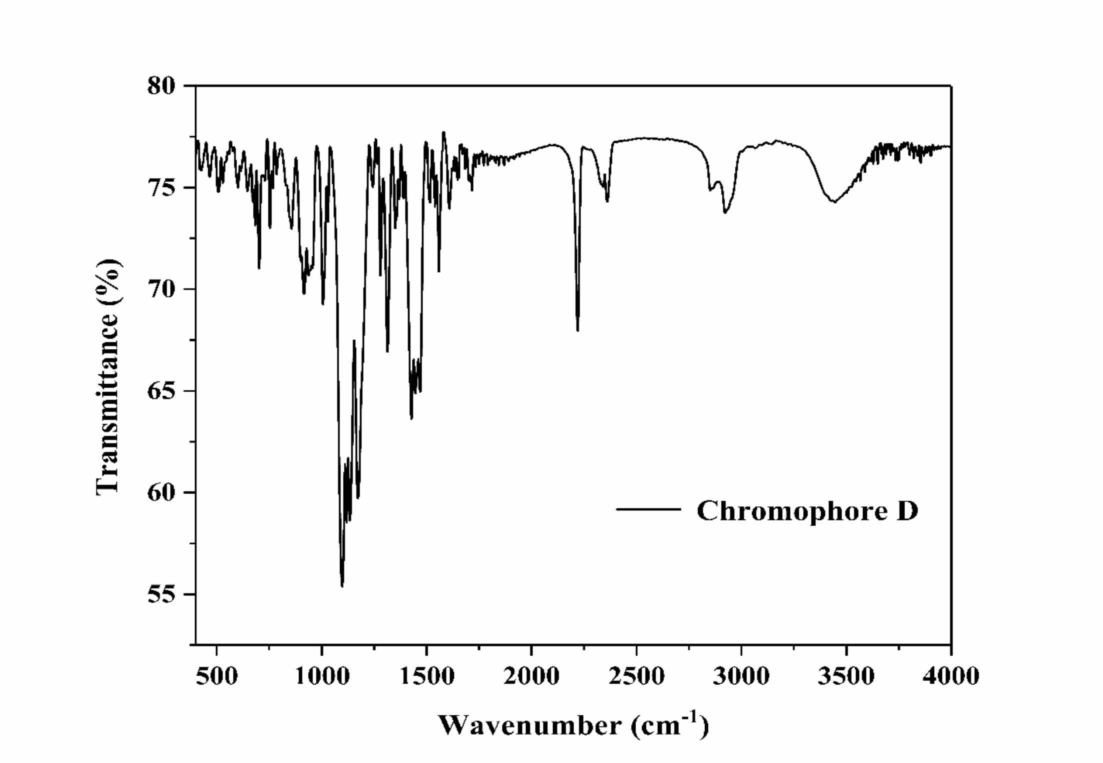

Figure S10. The FTIR spectra of the chromophore A, B, C and D.
